# Ribosomal RNA cleavage by the previously unidentified RelS–RelI toxin–antitoxin system controls growth of *Mycobacterium tuberculosis*

**DOI:** 10.1093/nar/gkag571

**Published:** 2026-06-22

**Authors:** Xue Han, Tom J Arrowsmith, Svetlana Karamycheva, Xibing Xu, Michèle Coddeville, Carine Pagès, Bertille Voisin, Claude Gutierrez, Olivier Neyrolles, Kira S Makarova, Tim R Blower, Pierre Genevaux

**Affiliations:** Laboratoire de Microbiologie et Génétique Moléculaires, Centre de Biologie Intégrative, Université de Toulouse, CNRS, Toulouse 31062, France; Department of Biosciences, Durham University, Stockton Road, Durham DH1 3LE, United Kingdom; Division of Intramural Research, National Library of Medicine, National Institutes of Health, 8600 Rockville Pike, Bethesda, MD 20894, United States; Laboratoire de Microbiologie et Génétique Moléculaires, Centre de Biologie Intégrative, Université de Toulouse, CNRS, Toulouse 31062, France; Laboratoire de Microbiologie et Génétique Moléculaires, Centre de Biologie Intégrative, Université de Toulouse, CNRS, Toulouse 31062, France; Laboratoire de Microbiologie et Génétique Moléculaires, Centre de Biologie Intégrative, Université de Toulouse, CNRS, Toulouse 31062, France; Institut de Pharmacologie et de Biologie Structurale, Université de Toulouse, CNRS, Toulouse 31077, France; Institut de Pharmacologie et de Biologie Structurale, Université de Toulouse, CNRS, Toulouse 31077, France; Institut de Pharmacologie et de Biologie Structurale, Université de Toulouse, CNRS, Toulouse 31077, France; Division of Intramural Research, National Library of Medicine, National Institutes of Health, 8600 Rockville Pike, Bethesda, MD 20894, United States; Department of Biosciences, Durham University, Stockton Road, Durham DH1 3LE, United Kingdom; New England Biolabs, 240 County Road, Ipswich, MA 01938, United States; Laboratoire de Microbiologie et Génétique Moléculaires, Centre de Biologie Intégrative, Université de Toulouse, CNRS, Toulouse 31062, France

## Abstract

Toxin–antitoxin (TA) systems use diverse strategies to control bacterial growth and represent attractive therapeutic targets to fight pathogens. *Mycobacterium tuberculosis*, the bacterium responsible for human tuberculosis, encodes one of the largest repertoires of TA systems. Here, we applied a bioinformatic pipeline to predict candidate TA systems in mycobacterial genomes and identified Rv2663–Rv2664 (RelS–RelI) as a previously undetected system in *M. tuberculosis*. We show that the RelS toxin is highly toxic and is inhibited by a unique antitoxin, RelI. The 1.70 Å X-ray crystallographic structure of RelS:RelI shows an unprecedented heterooctameric quaternary TA complex formed by paired tetramers. In each tetramer, RelS toxins are held at each end of a RelI antitoxin dimer. RelI binds across the putative catalytic center of RelS, resulting in occlusion of essential putative target-binding residues. Investigation of the toxic mechanism revealed that RelS is an atypical RelE/ParE-like RNase toxin that inhibits translation by targeting the 30S ribosomal subunit, specifically cleaving the 16S ribosomal RNA between positions C1520 and U1521, a unique site within the anti-Shine–Dalgarno (anti-SD) core region. This work further highlights the anti-SD region as a hot spot for RNase toxins and extends the arsenal of TA systems harnessed by this major pathogen.

## Graphical Abstract

**Graphical Abstract:**
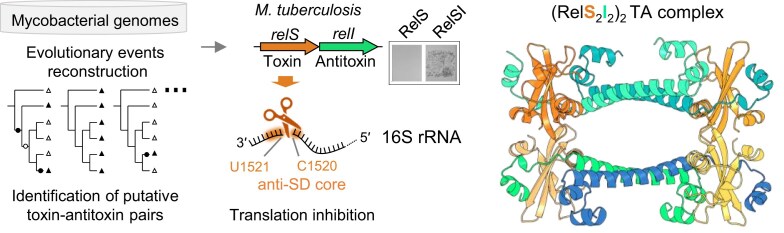

## Introduction

Toxin–antitoxin (TA) systems are stress-responsive elements that are widespread throughout bacterial and archaeal genomes, and on mobile genetic elements [1]. They are composed of a deleterious toxin and an antagonistic antitoxin, which inhibits toxin expression or activity. It is believed that in the absence of stress, the toxin is blocked by the antitoxin, and bacterial growth is not detectably affected. In the presence of stress, such as antibiotic exposure, phage infection, or plasmid loss, the equilibrium between the toxin and the cognate antitoxin can be dysregulated in favor of the toxin. In this case, the active toxin can target essential cellular processes or structures, mainly translation, DNA replication, metabolism, or the cell envelope, and cause growth inhibition or eventually cell death [1, 2]. Self-poisoning by active toxins was shown to have critical roles in defending bacteria against phage infection, in the maintenance of genomic regions or plasmids, and in bacterial virulence and antibiotic persistence [1, 3–6].

Tuberculosis (TB) is the leading cause of death due to a single infectious agent, namely the bacterium *Mycobacterium tuberculosis*. According to the World Health Organization, over 10.5 million people fell ill with TB in 2024, and 1.08 million died from the disease (www.who.int). The increasing occurrence of multidrug-resistant TB and the emergence of extensively or totally drug-resistant strains represents a major threat to public health and necessitates the development of new intervention strategies, including novel drugs and innovative host-targeted therapies. The genome of *M. tuberculosis* encodes >86 TA systems, representing ~4% of its genes and covering large sets of different TA families, including VapBC, MazEF, MenAT, HigBA, RelBE, ParDE, Res–Xre, DarTG, PezAT, and RHH–GNAT, as well as other less conserved or uncharacterized pairs [7]. So far, many toxins of *M. tuberculosis* were shown to be toxic when overexpressed in *M. tuberculosis, M. smegmatis*, or *Escherichia coli*, and TA operons are often induced under relevant stress conditions like hypoxia, macrophage engulfment, or antibiotics [8–10]. In agreement with such properties, it has been proposed that activated toxins could potentially regulate growth of *M. tuberculosis* and thus contribute to its success as a major human pathogen [10, 11]. Accordingly, *vapC22, vapC36, higB1, menT2, mazF3/6/9*, or *menT3/T4* mutants have been shown to be impaired for infection in different hosts [12–17]. Furthermore, the highly deleterious nature of certain toxins (often associated with the essentiality of their cognate antitoxin) has raised the possibility that new antibacterial properties carried by toxins might be used either to identify new TB drug targets or directly as self-poisoning antimicrobials, alone or in combination with standard antibiotic therapy [18, 19].

The genome of the *M. tuberculosis* reference strain H37Rv has been extensively examined for the presence of new TA systems using different methods [10, 20]. Most, if not all, of the novel putative TA systems discovered by these approaches have been tested *in vivo* in *M. tuberculosis, M. smegmatis*, or *E. coli*, and in some cases, their toxic mechanisms elucidated. In this work, we have analyzed a representative set of mycobacterial genomes to identify adjacent gene pairs with high gene gain estimates, a typical evolutionary feature of known TA systems, and identified *rv2663–2664* as a putative new TA operon in *M. tuberculosis*. We show that the putative toxin Rv2663 (now named RelS) is highly toxic in *M. tuberculosis* and that its deleterious effect is inhibited by co-expression of the putative antitoxin Rv2664 (now named RelI), indicating that RelS–RelI acts as a *bona fide* TA system in *M. tuberculosis*. We solved the crystal structure of the RelS:RelI TA complex and showed that it forms a novel heterooctameric structure composed of four RelS protomers with an RNase T1 fold similar to the BrnT toxin of *Brucella abortus* [21], bound to two RelI antitoxin dimers that have no structural homologs. Within the complex, RelI α1 helices sterically occlude the putative catalytic center of RelS, thus blocking toxin activity. Investigation of the RelS toxic mechanism revealed that RelS efficiently inhibits translation but does not cleave messenger RNA (mRNA). Instead, we found that RelS acts as a ribonuclease that specifically targets the 30S ribosomal subunit by cleaving the 16S ribosomal RNA (rRNA) at its extreme 3′ end, within the conserved anti-Shine–Dalgarno (anti-SD) region.

## Materials and methods

### Bacterial strains and culture conditions

*Escherichia coli* strains DH5α (Invitrogen), BL21(λDE3) AI (Novagen), DLT1900 [22], *M. smegmatis* mc^2^155 (strain ATCC 700084), and *M. tuberculosis* H37Rv [wild type (WT), ATCC27294] [23] have been described. The *M. tuberculosis* H37Rv △*(rv2663–rv2664)::ZeoR* mutant strain was constructed by allelic exchange using recombineering, as previously described [24]. In this case, ∼0.5-kb DNA fragments flanking the *rv2663–rv2664* operon were amplified by polymerase chain reaction (PCR) using primer pairs Rv2663-Am-Fw/Rv2663-Zeo-Am-Rv or Rv2664-Zeo-Av-Fw/Rv264-Av-Rv, and *M. tuberculosis* H37Rv genomic DNA as template. Zeocin-Resistance cassette (Zeo^R^) was PCR amplified using primer pair Zeo-Dir/Zeo-Rev and inserted between the two flanking regions of homology by a three-fragments fusion PCR with primer pair Rv2663-Am-Fw/Rv2664-Av-Rv. The resulting linear DNA was electroporated into *M. tuberculosis* H37Rv strain containing plasmid pJV53 (Kan^R^), as described [25]. *Mycobacterium tuberculosis* zeocin-resistant recombinant clones were selected, and the position of the insert into the genome was verified by PCR, using primers Rv2663-Am-Fw/Rv2664-Av-Rv, and two primers internal to the deletion, Rv2663-int-Rev and Rv2664-int-Fw.

*Escherichia coli* strains were grown at 37°C in LB supplemented, when necessary, with kanamycin (Km, 50 μg ml^−1^), ampicillin (Ap, 50 μg ml^−1^), streptomycin (Sm, 100 μg ml^−1^), isopropyl-β-D-thiogalactopyranoside (IPTG, 1 mM), L-arabinose (L-ara, 0.1% w/v), or D-glucose (glu, 0.2% w/v). *Mycobacterium smegmatis* mc^2^155 strains were grown at 37°C in LB supplemented with 0.05% v/v Tween 80 (Sigma–Aldrich) and, when necessary, with Km (10 μg ml^−1^) or Sm (25 μg ml^−1^). *Mycobacterium tuberculosis* strains were grown at 37°C in 7H9 medium (Middlebrook 7H9 medium, Difco) supplemented with 10% v/v oleic acid-albumin-dextrose-catalase (OADC, Difco) and 0.05% v/v Tween 80, or on complete 7H11 solid medium (Middlebrook 7H11 agar, Difco) supplemented with 10% v/v OADC. When necessary, Sm (25 μg ml^−1^), zeocin (Zeo, 25 μg ml^−1^), or anhydrotetracycline (Atc, 100 or 200 ng ml^−1^) were added [26].

### Plasmid constructs

Plasmids pGMC [27], pLAM12[25], pMPMK6 [28], p29SEN [29], and pET20b (Novagen) have been described. All the primers used to construct the plasmids are described in Supplementary Table S1, and a list of plasmids is provided in Supplementary Table S2.

To construct pGMC-gene plasmids expressing the 14 new putative toxin–antitoxin systems, namely rv2663, rv2664, AWB99_RS04530, AWB99_RS04535, Mycch_5153, Mycch_5154, BST38_RS17710, BST38_RS29380, BLW81_RS13130, BLW81_RS13135, AFA91_01 820, AFA91_01 825, Mycch_5672, and Mycch_5673 (Datasheet 1), 12 genes, excluding Rv2663 and Rv2664, were first synthesized and cloned into pUC57 by Genewiz (Azenta Life Sciences). The genes were PCR amplified using appropriate primers and cloned into linearized pGMC plasmid by homologous recombination using the In-Fusion HD Cloning Kit (Takara Bio). Genes rv2663 (relS) and rv2664 (relI) were PCR amplified from *M. tuberculosis* H37Rv genomic DNA using primers pGMC-Rv2663_Fw and pGMC-Rv2663_Rv, and primers pGMC-Rv2664_Fw and pGMC-Rv2664_Rv, respectively, and cloned into linearized pGMC plasmid by homologous recombination. To construct the plasmid pGMC-WSD-RelS featuring a weak Shine–Dalgarno sequence upstream of relS, QuikChange site-directed mutagenesis was utilized using primers pGMC-Rv2663_weakSD_Fw and pGMC-Rv2663_weakSD_Rv, with pGMC-RelS serving as the template. Specifically, the RBS1 region (AGGAAGACAGGCTGCCC), which corresponds to a strong Shine–Dalgarno sequence, was replaced with RBS4 (ACGAAGACAGGCTGCCC), corresponding to a weaker Shine–Dalgarno [30]. Plasmids pGMC-WSD-RelS with S6A, K9A, H10A, L25A, Y27A, E31A, Y32A, H33A, T46A, R48A, R69A, or K71A substitutions in RelS were also constructed by QuikChange site-directed mutagenesis using appropriate primers and pGMC-WSD-RelS as template. Plasmid pGMC-RelS–RelI was constructed as follows. The rv2663–2664 operon was PCR amplified from the *M. tuberculosis* H37Rv genome using primers pGMC-63_64_Fw and pGMC-63_64_Rv and cloned into linearized pGMC plasmid by homologous recombination. Plasmids pGMC-RelS–RelI with T5A, D6A, D8A, W10A, D12A, H22A, R29A, E35A, E43A, or R69A substitutions in RelI were constructed by QuikChange site-directed mutagenesis using appropriate primers and pGMC-RelS–RelI as template. Plasmids pGMC-RelS–RelI with double D6A/D8A substitutions or triple D6A/D8A/D12A substitutions in RelI were constructed by QuikChange site-directed mutagenesis using Rv2664_D6A/D8A_Fw and Rv2664_D6A/D8A_Rv, Rv2664_D6A/D8A/D12A_Fw and Rv2664_D6A/D8A/D12A_Rv and pGMC-RelS–RelI^D6A^ as template. Plasmids pGMC-RelS-∆(2–24)RelI, pGMC-RelS-∆(26–54)RelI, and pGMC-RelS-∆(57–84)RelI generating N-terminal (2–24 aa), middle helix (26–54 aa), and C-terminal (57–84 aa) deletion variants of RelI were constructed by PCR amplification using primers pGMC-63_64_Del_N24aa_Fw and pGMC-63_64_Del_N24aa_Rv, pGMC-63_64_N25aa-C30aa_FW and pGMC-63_64_N25aa-C30aa_RV, pGMC-63 + 64_Del_C28aa _Fw and pGMC-63 + 64_Del_C29aa _Rv, with pGMC-RelS–RelI as template.

To construct plasmids pLAM12-RelS, pLAM12-RelI, pLAM12- *AWB99_RS04530*, and pLAM12- *AWB99_RS04535, relS, relI, AWB99_RS04530*, and *AWB99_RS04535*, were PCR-amplified using pGMC-RelS, pGMC-RelI, pGMC-*AWB99_RS04530*, and pGMC-*AWB99_RS04535* as templates, respectively. These were cloned as NdeI/EcoRI fragments (*relS, relI, AWB99_RS04530*, and *AWB99_RS04535*) into NdeI/EcoRI digested pLAM12. Plasmids pLAM12-RelI with D6A, D8A, W10A, D12A, H22A, R29A, E35A, E43A, or R69A substitutions of RelI were constructed by QuikChange site-directed mutagenesis using appropriate primers and pLAM12-RelI as template. Plasmids pLAM12-RelI with double D6A/D8A substitutions or triple D6A/D8A/D12A substitutions of RelI were constructed by QuikChange site-directed mutagenesis using Rv2664_D6A/D8A_Fw and Rv2664_D6A/D8A_Rv, Rv2664_D6A/D8A/D12A_Fw and Rv2664_D6A/D8A/D12A_Rv, and pLAM-RelI^D6A^ as template. Plasmids pLAM12-∆(2–24)RelI, pLAM12-∆(26–54)RelI, and pLAM12-∆(57–84)RelI generating N-terminal (2–24 aa), middle helix(27–54 aa), and C-terminal (57–84 aa) deletion variants of RelI were constructed as follows. RelS–RelI was PCR amplified using primers pLAM12-Rv2664-delN2-24 and pLAM12-Rv2664-infu-RV, pLAM12-Rv2664-infu-Fw and pLAM12-Rv2664-infu-RV, and pLAM12-Rv2664-infu-Fw and pLAM12-Rv2664-delC57-84 with pGMC-RelS-∆(2–24)RelI, pGMC-RelS-∆(26–54)RelI, and pGMC-RelS-∆(57–84)RelI as templates. RelS–RelI deletions were then cloned into linearized pLAM12 plasmid by homologous recombination.

Plasmid pMPMK6-RelS was constructed as follows. *relS* was PCR amplified from pGMC-RelS using primers Rv2663 EcoRI-For and Rv2663 HindIII-Rev, and cloned as EcoRI/HindIII fragments into EcoRI/HindIII digested pMPMK6.

To obtain plasmid p29SEN-RelI, *rv2664* was PCR amplified from the pGMC-RelI using primers Rv2664-p29SEN-ligation-Fw and Rv2664-p29SEN-ligation-Rv, and cloned as EcoRI/HindIII fragments into EcoRI/HindIII digested p29SEN.

To generate pET20b-RelS, *rv2663* was PCR amplified from pGMC-RelS using primers pET20b-Rv2663_Liga_Fw and pET20b-Rv2663_Liga_Rv, and cloned as NdeI/XhoI fragments into NdeI/XhoI digested pET20b. Plasmid pET20b-RelS^H10A^ was constructed by QuikChange site-directed mutagenesis using pET20b-RelS as a template. To construct pET15b-RelI and pET15b-∆(2–24)RelI, *rv2664* and truncated *rv2664* were PCR amplified from pLAM12-RelI and pLAM12-∆(2–24)RelI using primers 15b- RelI NdeI For, 15b- RelI del 2–24 For, and 15b- RelI BamHI Rv, and cloned as NdeI/BamHI fragments into NdeI/BamHI digested pMPMK6.

Plasmids pTRB686 (pTRB550:His-SUMO-RelS), pTRB715 (pTRB550:His-SUMO-RelS H10A), pTRB695 (pTA100:RelI), and pTRB743 (pTA100:RelI∆2–24) were synthesized by Genscript Biotech Ltd.

All the plasmids constructed in this work have been verified by sequencing.

### Identification of new putative TA systems in mycobacteria

Fifty completely sequenced genomes from *Mycobacteriaceae* family were selected (Datasheet 1) to build 16 195 clade-specific clusters of orthologous genes (csCOGs), and the phylogenetic tree for this genome set was reconstructed based on 16S rRNA sequences using the previously described procedure [31]. Given the 16S RNA tree and presence/absence patterns for csCOGs, the GLOOME program [32] was used to reconstruct basic evolutionary events, such as gains and losses, and based on this data, the number of gene gains was calculated for each csCOG. These estimates were assigned to each gene in each genome, and conserved adjacent pairs of genes with gene number estimates greater or equal than 2.5 were analyzed further using sequence analysis. Initially all proteins were annotated using PSI-BLAST [33] with an *E*-value threshold of 0.001 and compositionally based statistics turned off against the conserved domain database (CDD) [34]. Proteins in the selected gene pair with high gene gain estimates without CDD annotation were additionally analyzed using more sensitive HHpred searches with default parameters against PDB, PFAM, and CDD HMM profiles [35]. Muscle5 [36] with default parameters was used for protein multiple alignment construction. The phylogenetic tree was reconstructed using FastTree program (WAG evolutionary model, gamma-distributed site rates) [37]. The same program was used to calculate support values. Proteins in the originally identified csCOGs were discarded from further analysis if (i) they were functionally annotated proteins based on assignments to CD and PFAM databases [38]; (ii) they were predicted as membrane by TMHMM program [39] or secreted by SignalP program [40]; and (iii) they were >300 aa. Their respective gene neighbours were also discarded. The resulting 50 gene pairs (101 adjacent csCOGs) were analysed in more detail by examining their gene neighbourhood and using HHpred search with default parameters against PDB, PFAM and CDD HMM profiles to identify remote sequence similarity [35]. Based on this analysis, seven pairs were selected for experimental validation.

### Protein expression and purification

To purify RelS WT and RelS^H10A^, BL21(λDE3) AI strain was transformed with pET20b-RelS or pET20b-RelS^H10A^ and grown to an OD_600_ of ~0.4 at 37°C. 0.2% v/v of L-ara inducer was added, and the culture was immediately incubated overnight at 22°C. Cultures were centrifuged at 5000 × *g* for 10 min at 4°C, and pellets were washed with 3 ml lysis buffer and resuspended in 7 ml lysis buffer (10 mM Tris-HCl pH 7.4, 150 mM NaCl, 10 mM imidazole supplemented with one EDTA-free Protease Inhibitor tablets (Roche) and 500 units of benzonase (Sigma-Aldrich) in a final volume of 20 ml). Cells were lysed using a One-shot cell disrupter at 1.5 Kbar (One shot model, Constant Systems Ltd) and incubated for 2 h on ice. Lysates were centrifuged for 30 min at 30 000 × *g* at 4°C, and the resulting supernatants were gently mixed with Ni-NTA Agarose beads (Qiagen) pre-equilibrated with lysis buffer at 4°C for 30 min in a 10 ml poly-prep column (Bio-Rad). The column was then stabilized for 10 min at 4°C, washed six times with 10 ml of lysis buffer and 10 ml of wash buffer (10 mM Tris–HCl, pH 7.4, 150 mM NaCl, 75 mM imidazole), and RelS WT and RelS H10A proteins were eluted with 400 µl elution buffer (10 mM Tris–HCl, pH 7.4, 150 mM NaCl, 200 mM imidazole). Imidazole was removed using a PD MiniTrap G-25 column (Cytiva) equilibrated with 8 ml equilibration buffer (10 mM Tris–HCl, pH 7.4, 150 mM NaCl). Proteins were concentrated using Vivaspin® 6 columns with 5000 MWCO PES membrane (Sartorius) and used directly in subsequent experiments.

To purify the RelS:RelI complex for crystallographic studies, *E. coli* DH5α were co-transformed with plasmids pTRB686 (encoding His_6_-SUMO-RelS) and pTRB695 (encoding RelI) and grown overnight at 37°C with 150 rpm shaking. Cultures were then re-seeded 1:100 v/v into 2 l baffled flasks and grown to an OD_600_ of 0.55. Cells were then supplemented with 0.2% v/v L-ara and 1 mM IPTG to facilitate induction of toxin and antitoxin expression, respectively, with cultures incubated overnight at 17.5°C. The following morning, cells were harvested and resuspended in ice-cold A500 buffer (20 mM Tris–HCl, pH 7.9, 500 mM NaCl, 30 mM imidazole, 10% v/v glycerol), lysed by sonication (45% amplitude, 10 s pulse intervals, 2 min), and clarified by centrifugation (40 000 × *g*, 20 min, 4°C). Clarified cell lysate was loaded onto a pre-equilibrated 5 ml HisTrap column and washed with 50 ml of A500. Proteins were then eluted from the column with 25 ml B500 (20 mM Tris–HCl, pH 7.9, 500 mM NaCl, 250 mM imidazole, 10% v/v glycerol), with eluate subsequently dialyzed overnight against A100 (20 mM Tris–HCl, pH 7.9, 100 mM NaCl, 10 mM imidazole, 10% v/v glycerol) alongside 250 µl of 2 mg ml^−1^ human sentrin/SUMO-specific protease 2 (hSENP2) to facilitate cleavage of the His_6_-SUMO tag from RelS. The following day, this sample was loaded onto a second HisTrap column configured directly above a 5 ml HiTrap Q anion-exchange column. Both columns were washed with 25 ml A100 before transferring the Q column to an ÄKTA™ Pure FPLC for gradient elution of target proteins. Proteins were eluted by anion exchange chromatography using a salt gradient from 100% A100 to 30% C1000 (20 mM Tris–HCl, pH 7.9, 1 M NaCl, 10% v/v glycerol). The purity of peak chromatographic fractions was confirmed by sodium dodecyl sulfate–polyacrylamide gel electrophoresis (SDS–PAGE). Fractions containing target proteins were then pooled and concentrated by centrifugation using the appropriate MWCO Vivaspin concentrator (Sartorius), then further purified by size-exclusion chromatography (SEC) using a Superdex™ 75 increase 10/300 GL SEC column. Samples were loaded onto a 100 μl capillary loop and applied to the column by flooding the loop with analytical SEC buffer (20 mM Tris–HCl, pH 7.9, 150 mM NaCl). The final sample was submitted to the Durham University In-House Mass Spectrometry facility for confirmation of purity prior to crystallographic studies. HigB1 [41], RelE1, and RelE3/YoeB [42] proteins were purified as described.

### Western blot analysis

Strain BL21(λDE3) AI transformed with pET15b vector, pET15b-RelI, or pET15b-∆(2–24)RelI was grown to an OD_600_ of ∼0.4 at 37°C. 0.2% L-ara was added, and the cultures were incubated overnight at 22°C. Cultures were centrifuged at 5000 × *g* for 10 min at 4°C, pellets were washed with 3 ml lysis buffer (25 mM equimolar solution of Na_2_HPO_4_/NaH_2_PO_4_; 200 mM NaCl; 20 mM imidazole pH 8.0) and resuspended in 7 ml lysis buffer supplemented with one ethylenediaminetetraacetic acid (EDTA)-free Protease Inhibitor tablets (Roche, to 20 ml) and benzonase 25 U ml^−1^ (Sigma–Aldrich). Lysis was performed using the One-shot cell disrupter at 1.5 Kbar (One shot model, Constant Systems Ltd), and lysates were centrifuged for 30 min at 30 000 × *g* at 4°C. Proteins (2 μl of lysate supernatant; 5.5 μl of lysis buffer; 2.5 μl of 4 × SDS loading buffer) were then separated on Mini-Protein TGX gels (BioRad) by SDS–PAGE and transferred to polyvinylidene difluoride membrane (Bio-Rad) using the Trans-Blot^®^ TurboTM transfer system (Bio-Rad). Membrane was blocked for 1 h at room temperature (RT) in 5% (w/v) nonfat dry milk in phosphate buffered saline containing 0.05% (v/v) Tween 20. Primary antibody used in this study was anti-His antibody (QIAGEN, dilution 1:1000). Horseradish peroxidase-conjugated mouse IgG (Promega, 1:2500) was used as a secondary antibody. Blots were developed by chemiluminescence using Clarity Western ECL substrate (Bio-Rad) with the ChemidocTM Touch imaging system (Bio-Rad) and analyzed with the Image Lab software (Bio-Rad).

### 70S ribosomes and 30S ribosomal subunit purification

Ribosomes were purified from 1 L of *M. smegmatis* mc^2^155 culture grown at 37°C in LB. When the culture reached an OD_600_ of 1.0, cells were pelleted by centrifugation at 5000 × *g* for 10 min at 4°C and washed with 14 ml lysis buffer (20 mM Tris–HCl, pH 7.5, 10.5 mM MgO(Ac)_2_, 100 mM NH_4_Cl, 0.5 mM EDTA, and 3 mM 2-mercaptoethanol), resuspended in 16 ml lysis buffer and lysed using a One-shot cell disrupter at 1.3 Kbar three times. The lysate was then clarified by centrifugation at 20 000 × *g* for 10 min and 25 000 × *g* for 1 h at 4°C. Next, the supernatant was layered 1:1 (v:v) over a high-salt sucrose cushion buffer (20 mM Tris–HCl, pH 7.5, 10.5 mM MgO(Ac)_2_, 500 mM NH_4_Cl, 0.5 mM EDTA, 3 mM 2-mercaptoethanol, 1.1 M sucrose) in 6 Quick-Seal Centrifuge Tubes (Beckman Coulter, 5.1 ml). After ultracentrifugation at 100 000 × *g* for 22 h at 4°C, the resulting ribosome pellets were washed twice with 100 µl storage buffer (10 mM Tris–HCl, pH 7.5, 10.5 mM MgOAc, 60 mM NH_4_Cl, 3 mM 2-mercaptoethanol) and resuspended in 8 µl of storage buffer overnight at 4°C. Ribosomes were flash-frozen in liquid nitrogen and stored at −80°C until further use. To dissociate the 70S ribosome to respective subunits, 30S and 50S, the concentration of MgOAC was reduced from 10.5 to 5 mM, and KCl was added to a concentration of 500 mM, followed by incubation on ice for 1 h 30 min. The ribosomes and subunit mixture was layered on a 10%–30% sucrose gradient prepared in Tp gradient buffer (50 mM Tris–HCl, pH 7.5, 5 mM MgCl_2_, 500 mM KCl), and centrifugation was carried out at 186 000 × *g* for 3.2 h. The peaks corresponding to 50S and 30S were collected and concentrated with Vivacon® 2 centrifugal devices separately. The concentration was estimated by measuring the absorbance at 260 nm. Aliquots of subunits were flash-frozen in liquid nitrogen and stored at −80°C until further use.

### Cell-free transcription–translation system *in vitro*

Cell-free transcription/translation *in vitro* assays were performed as described [41, 43]. Briefly, DNA of *cspA* (*rv3648c)* 204 bp and *gfp* 717 bp were amplified by PCR using primers containing the T7 polymerase promoter and terminator and added at a final concentration of 20 ng μl^−1^ to the *E. coli* PURE system. Protein synthesis was performed at 37°C for 2 h in the presence of 0.6 μCi μl^-1^ of [^35^S]-methionine with or without toxins (5 µM). The reaction was stopped by placing on ice, and samples were then separated by SDS/PAGE on 4%–20% Mini-Protean TGX gels (Bio-Rad) for 30 min at 200 V. Gels were fixed in 10% acetic acid/40% methanol (v/v) for 30 min, and proteins were visualized using a Typhoon phosphorimager (GE Healthcare) and Multigauge software (Fuji).

*Mycobacterium smegmatis* hybrid *in vitro* transcription/translation system was performed as follows. Purified *M. smegmatis* ribosomes were added to PURExpress® Δ Ribosome Kit (NEB) at a final concentration of 2.5 µM. Freshly purified toxins (5 µM RelS WT; 5 µM RelS H10A; 10 µM RelE1;10 µM HigB1, and 10 µM YoeB) were added to the system. DNA of *cspA* (*rv3648c)* 204 bp and *gfp* 717 bp were amplified as previously described and T7-DNA templates were added at a final concentration of 20 ng μl^−1^ to the *M. smegmatis* transcription/translation system. Similar experiments were also performed by directly adding purified mRNA. In this case, mRNA of *cspA* and *gfp* were *in vitro* transcribed with T7 RNA polymerase (NEB), purified, and then added to cell-free *M. smegmatis* transcription/translation reactions at a final concentration of 60 ng μl^−1^. Protein synthesis was performed at 37°C for 2 h in the presence of 0.6 µCi µl^-1^ of [^35^S]-methionine, and the reaction was stopped by placing on ice. Protein synthesis was visualized as described earlier for the *E. coli* PURE assay.

In addition to the monitoring of protein synthesis, total RNA from *in vitro* translation reactions were extracted for subsequent experiments, including mRNA reverse transcription, northern blotting assay, and RNA sequencing. In this case, cold DEPC-H_2_O (pH 7.0) was added to each *in vitro* translation reaction to a total volume of 100 µl, followed by 300 µl of TRIzol (Sigma–Aldrich) and 100 µl of cold chloroform. After mixing for 30 s and centrifugation at 20 000 ×* g* at 4 °C for 15 min, the aqueous phase was transferred to a new centrifuge tube, and 100 µl of isopropanol was added to precipitate the RNA. RNA was washed twice with 75% ethanol, then dissolved in DEPC-H_2_O and stored at −80°C until further use.

### Primer extension

Reverse transcription experiments were performed as follows. Two micrograms of purified RNA from the *M. smegmatis* transcription/translation extracts, 0.05 µM [^32^P]-labeled *cspA* extension primer, and 1 mM dNTPs were mixed in a 10 µl volume, incubated at 65°C for 5 min, and chilled on ice for 2 min. Finally, 10 µl of 2× buffer [mix 4 µl 5× ProtoScript II RT (NEB), 2 µl 0.1M DTT, 8 units RNasin® Plus Ribonuclease Inhibitor (Promega), and 200 units of ProtoScript II RT Enzyme (NEB) in 10 µl] was mixed and incubated at 48°C for 1 h. The resulting complementary DNA (cDNA) was mixed with RNA loading dye, loaded on a 6% polyacrylamide gel containing 7 M urea, separated at 200 V for 2 h 30 min, and revealed by autoradiography using Typhoon phosphorimager (GE Healthcare) and Multigauge (Fuji Film). Note that [^32^P]-labeling of the primer was performed using T4 Polynucleotide Kinase (10U, NEB) at 37°C for 1 h in the presence of extension primer (0.5 µM final concentration) and 2.5 µCi µl^−1^ of ATP, [γ-^32^P]. The labeled primer was purified with Bio-Spin® 6 Columns (Bio-Rad).

### RelS overexpression *in vivo*

RelS was overexpressed in both *M. smegmatis* and *M. tuberculosis*, and total RNA were extracted for subsequent experiments, including northern blotting assay and RNA sequencing. In this case, *M. smegmatis* was transformed with pGMC, pGMC-WSD-RelS, or pGMC-WSD-RelS H10A and grown for 3 days at 37°C. Cell cultures were transferred to fresh LB medium and grown at 37°C until reaching an OD_600_ of 0.1. Atc (100 ng ml^−1^) inducer was added to the cultures, and equivalent amounts of cells were pelleted by centrifugation at 3500 × *g* for 10 min at 4°C at different time points after incubation at 37°C for preparation of total RNA. For each sample, pellets were resuspended in 1 ml cold TRIzol and transferred to 2 ml Safe-Lock tubes with 35–50 mg acid-washed glass beads [425–600 μm (30–40 U.S. sieve), Sigma–Aldrich]. Cells were disrupted using a bead-beater disrupter (Precellys® 24 Touch, Bertin Technologies) for a total of 6 min (3 × 1 min ON, 30s OFF, 1 min ON). The lysate was centrifuged at 20 000 × *g* at 4 °C for 2 min, with the resultant supernatant then transferred to a new RNase-free Eppendorf tube. Samples were supplemented with 400 µl of cold chloroform and gently shaken for 30 s, then immediately incubated on ice for 15 min. Each sample was then centrifuged at 20 000 × *g* for 20 min at 4°C, and the upper aqueous phase was transferred into a new RNase-free Eppendorf tube and supplemented with 500 µl of cold isopropanol, then mixed immediately and incubated at −20 °C overnight to precipitate RNA. Finally, isolated RNA samples were washed twice with 75% ethanol, then dissolved in DEPC-H_2_O and stored at −80°C until further use.

In the case of *M. tuberculosis* H37Rv △*rv2663–2664*, the strain was transformed with pGMC, pGMC-WSD-RelS, or pGMC-WSD-RelS H10A and grown at 37°C until OD_600_ reached 0.4. Atc (200 ng ml^−1^) was added, and equivalent amounts of cells were pelleted by centrifugation at 3500 × *g* for 10 min at 4 °C after 0, 4, or 24 h. Cell pellets were resuspended in 1 ml of TRIzol, and cells were disrupted using bead-beater disrupter. Lysates were centrifuged for 2 min at 20 000 × *g* at 4 °C, and TRIzol extracts were collected and conserved for at least 48 h at −80°C before being transferred out of the BSL3 laboratory for further total RNA isolation as described for *M. smegmatis* RNA preparation.

### Northern blotting assay

Northern blotting experiments were performed as described [44]. Briefly, equal amounts of total RNA (usually 4 μg for analysis of rRNA *in vivo* or 2 μg for rRNA *in vitro M. smegmatis* transcription/translation system) were mixed with five volumes of glyoxal loading buffer [prepared by mixing the following: 6 ml DMSO, 2 ml deionized glyoxal, 1.2 ml 10× BPTE (see below), 600 μl 80% glycerol, and 40 μl 10 mg ml^−1^ ethidium bromide]. The samples were heated for 1 h at 55°C, and RNAs were separated by electrophoresis on 1.2% agarose gels in 1× BPTE running buffer (100 mM PIPES, 300 mM Bis-Tris, 10 mM EDTA). After electrophoresis, the gels were (i) rinsed 10 min with ultrapure MilliQ H_2_O, (ii) soaked 20 min at RT in 75 mM NaOH with gentle shaking to partially hydrolyze RNA, (iii) rinsed 2 × 5 min with ultrapure MilliQ H_2_O, (iv) soaked 2 × 15 min at RT in (0.5 M Tris–HCl, pH 7.4, 1.5 M NaCl) with gentle shaking to neutralize the pH, (v) rinsed 10 min with ultrapure MilliQ H_2_O, and (vi) soaked 2 × 10 min at RT in 10× saline-sodium citrate (SSC; 1.5 M NaCl, 0.15 M trisodium citrate, pH 7.0) with gentle shaking. RNAs were then transferred over night to Amersham Hybond-N+ membranes (GE Healthcare) by capillary action with 10× SSC transfer buffer. Membranes were then exposed to 0.125 joules of 365 nm UV rays to crosslink RNA on the membranes. Membranes were then hybridized with [^32^P]-labeled oligonucleotide probes using the ROTI®Hybri-Quick buffer (ROTH). Radioactive membranes were exposed to PhosphorImager screens, and signals were measured using a Typhoon imager (GE Healthcare). The sequences of the probes used to detect (pre-)rRNA are described in Supplementary Table S1.

### Incubation assay of RelS with different states of 16S rRNA *in vitro*

16S rRNA cleavage by RelS in the presence of purified 70S, 30S ribosomal subunits or free 16S rRNA was performed as follows. 2.5 µM of 70S ribosome, 2 ng of 30S subunits, and 2 ng of RNA extracted from *M. smegmatis* transcription/translation system were incubated with or without 10 µM of toxin, respectively, for 2 h at 37°C. Then RNA was extracted using TRIzol and isopropanol precipitation. RNA was dissolved in DEPC-H_2_O and kept at −80°C until further use.

### RNase H digestion assay and RNA analysis

RNA extracts from (i) *in vitro* transcription–translation assay, (ii) *in vivo M. smegmatis* with or without RelS overexpression for 1 or 3 h, and (iii) *in vitro* 30S subunits/16S rRNA incubation assay were first incubated with an RNA/DNA/RNA reverse probe hybridizing in the 180 nt from 3′ end of 16S rRNA, and then RNase H (NEB) was used to digest the RNA samples to obtain an RNA fragment ~180 nt from the 3′ end of 16S rRNA, which could subsequently be sequenced. In this case, for each RNA sample, 2 µg of RNA (8 µl) were denatured at 95°C for 5 min with the RNA/DNA/RNA reverse probe (16S_RNaseH_ probe_1: 5′-ACGUAUUCACCGCAGCGTTGCUGAUCUGCGAUUAC-3′; 1 µl at 100 mM). After annealing by cooling down to RT for 10 min, 9 µl of H_2_O was added and mixed well before transferring 9 µl to a new 1.5 ml tube. The RNA mixture was diluted to 30 µl with a reaction mix containing 1× RNase H reaction buffer, 66 µM DTT, 20 U RNasin® Plus Ribonuclease Inhibitor, and 50 U RNase H (New England Biolabs) or an equal volume of water for the control group, and incubated at 37°C for 30 min. The reaction was then blocked by addition of 0.3 M sodium acetate, pH 5.2, and 0.2 mM EDTA, and the RNA were recovered by ethanol precipitation after phenol-chloroform-isoamylalcohol (25:24:1) extraction. RNAs were then separated on a 6% polyacrylamide gel (19:1) in 1× Tris-Borate-EDTA (TBE) buffer containing 7 M urea. RNA was stained with SYBR^TM^ Safe (Invitrogen) diluted 10 000-fold in 1× TBE buffer for 20 min and visualized by UV epi-illumination.

### 16S rRNA 3′ end library preparation

3′-OH RNA-seq library was constructed as follows. RNA was purified from RNA gels after RNase H digestion assay (described earlier). Gel slices of RNase H-treated RNA encompassing the 120–225 nt regions were soaked in 350 µl RNA elution buffer (0.3 M NaOAC, 0.1 mM EDTA, 0.1% SDS) overnight to elute RNA from the gels. RNA samples were precipitated with ethanol and glycogen (20 µg ml^−1^) at −20 °C overnight, dephosphorylated by T4 Polynucleotide Kinase (NEB), and ligated to the adenylated 3p-v4 adaptors. Reverse transcription was performed with SuperScript IV Reverse Transcriptase (Thermo Fisher Scientific) using barcode primers. Finally, PCR amplification was performed with primers M.smeg_16SrRNA_RNAseq_3 and A-PE-PCR10 (after five cycles, the program was paused and B_i7RPI1_CGTGAT, B_i7RPI2_ACATCG or B_i7RPI3_GCCTAA was added) using Q5 Polymerase Hot-Start. The library was sequenced by DNBSEQ-G400RS High-throughput Sequencing Set (PE150) in BGI Genomics (Poland). Primers used for the construction of 16S rRNA 3′end libraries are described in Supplementary Table S1, and raw sequencing data are provided in Datasheet 1.

### Protein crystallization and structure determination

The purified RelS–RelI complex was concentrated to 12 mg ml^−1^ in Crystal buffer (20 mM Tris–HCl, pH 7.9, 150 mM NaCl, 2.5 mM DTT), and crystallization screens were performed using a Mosquito Xtal3 robot (SPT Labtech) using the sitting-drop method, with 2:1 and 1:1 ratios of protein to mother liquor tested for each condition screen. RelS:RelI formed a small cuboid crystal in condition B1 (0.1 M sodium acetate, pH 5.5, 40% v/v MPD) of Clear Strategy II HT-96 eco screen (Molecular Dimensions). To harvest crystals for subsequent structural determination, 20 µl screen condition was mixed with 20 µl of cryo buffer (25 mM Tris–HCl, pH 7.9, 187.5 mM NaCl, 3.125 mM DTT, and 80% glycerol), then added to the protein crystal drop at a 1:1 v/v ratio. Crystals were then immediately extracted from the drop using the appropriately sized nylon loop and transferred to a unipuck immersed in liquid N_2_.

Diffraction data were collected at Diamond Light Source on beamline I24 (Table 1). Two 360⁰ datasets were collected at 0.9795 Å and merged using iSpyB (Diamond Light Source). Data were processed using AIMLESS from CCP4 [45] to corroborate spacegroups. The structure was solved *ab initio* and further built using REFMAC in CCP4 [46], then iteratively refined and built using COOT [47] (Ramachandran statistics; 99.83% favored, 0.17% allowed, 0.00% outliers). The quality of the final model was assessed using COOT and the wwPDB validation server [48]. Structural figures, including alignments and superpositions, were generated using PyMol (Schrödinger).

**Table 1.** Data collection and refinement statistics

RelS–RelI heterooctamer	
PDB ID code	9SDC
Number of crystals	1
Beamline	Diamond I24
Wavelength, Å	0.9795
Resolution range, Å	68.78–1.7 (1.72–1.7)
Space group	P 2_1_2 _1_2_1_
Unit cell	
*a b c* (Å)	67.854 76.955 137.551
*α β γ* (°)	90 90 90
Total reflections	153 176 (8140)
Unique reflections	80 001 (4187)
Multiplicity	1.9 (1.9)
Completeness (%)	100.0 (100.0)
Mean I/sigma(I)	6.8 (0.3)
*R*_merge_	0.049 (0.910)
*R*_meas_	0.069 (1.287)
CC_1/2_	0.998 (0.373)
*R*_work_	0.1974 (0.4011)
*R*_free_	0.2272 (0.3869)
No. of non-hydrogen atoms	5256
Macromolecules	4808
Solvent	448
Protein residues	611
RMSD (bonds, Å)	0.006
RMSD (angles, °)	0.77
Ramachandran favoured (%)	99.83
Ramachandran allowed (%)	0.17
Ramachandran outliers (%)	0.00
Average B-factor	31.83
Macromolecules	31.11
Solvent	39.56
Values in parentheses are for highest-resolution shell.	

## Results

### Identification of novel putative TA systems in mycobacterial genomes

**Figure 1.**
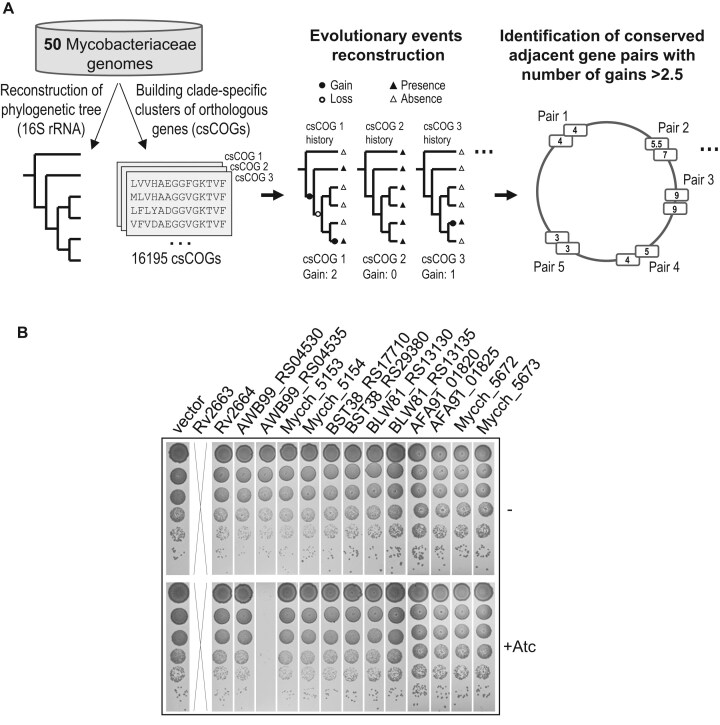
Identification and *in vivo* assessment of putative TA systems in mycobacteria. (**A**) Computational pipeline for identification of conserved adjacent gene pairs with high number of gene gain estimates. Fifty completely sequenced genomes from *Mycobacteriaceae* family were selected to build csCOGs, and the phylogenetic tree for this genome set was reconstructed based on 16S rRNA sequences. Given the tree and presence/absence patterns for 16 195 csCOGs, the GLOOME program was used to reconstruct basic evolutionary events, such as gains and losses. Once the evolutionary events were reconstructed, for each csCOG, the number of gains was calculated. For each gene in each genome, the csCOG and respective gain number estimates. Conserved adjacent pairs of genes for which csCOG with high gene number were analyzed further using sequence analysis and comparative genomic approaches as described in “Materials and methods” section. (**B**) Toxicity assay of the 14 putative TA genes expressed in *M. smegmatis. Mycobacterium smegmatis* transformed with pGMC-vector (−) or with each putative TA gene was serially diluted and spotted on LB agar plates with or without Atc inducer at 100 ng ml^−1^. No viable transformant could be obtained with pGMC-Rv2663. Plates were incubated 3 days at 37°C. Data are representative of at least three independent experiments.

To search TA system candidates, we explored a previously reported observation, that TA and many defense systems are prone to high gene flux (gains and losses) during evolution [49–51]. Thus, we obtained estimates of gain and loss for csCOGs constructed for 50 selected representative mycobacterial genomes (Datasheet 1). Subsequent mapping of gene gain estimates to all genes in each genome identified adjacent gene pairs where both genes have a gain estimate >2.5 [31, 52]; Fig. 1A). We then further analyzed these pairs of genes using sensitive sequence similarity searches and focused on those that contain at least one gene not associated with known TA systems (Datasheet 1). Seven new putative TA pairs (Datasheet 1) were selected for further experimental investigation on the basis of having (i) one of the proteins similar to a DNA-binding protein (thus potentially an antitoxin) and one unknown protein or a protein distantly related to known toxin (possibly the cognate toxin), or (ii) a pair with both genes unknown. Of note, this analysis revealed the unexpected presence of a new putative TA pair in the genome of *M. tuberculosis*, namely Rv2663–Rv2664.

The 14 genes were first separately cloned onto an integrative pGMC vector under the control of an Atc-inducible promoter and tested for their toxicity when overexpressed in *M. smegmatis* as host (Fig. 1B). The results show that only two out of 14 of the tested clones were toxic, namely the putative toxin AWB99_RS04535 from *M. confluentis* (also named *Mycolicibacterium confluentis)* and the putative toxin Rv2663 from *M. tuberculosis*. Note that in the case of Rv2663, we were not able to obtain viable transformants of *M. smegmatis*, even when cells were directly diluted and plated after recovery from electroporation (Fig. 1B, lane 2, see below). AWB99_RS04535 was not considered further because it is a predicted HEPN (higher eukaryotes and prokaryotes nucleotide-binding domain) toxin and AWB99_RS04530 is a nucleotidyltransferase, putative antitoxin, likely comprising a distant variant of a well-characterized type IV MNT-HEPN (minimal nucleotidyltransferase) TA system [53, 54]. Accordingly, AWB99_RS04535 toxicity in *M. smegmatis* was indeed inhibited by coexpression of AWB99_RS04530 (Supplementary Fig. S1). In contrast, the predicted Rv2664 antitoxin for Rv2663 did not show any significant sequence similarity to known antitoxins (Datasheet 1).

### Rv2663–2664 (RelS–RelI) is a *bona fide* TA system in *M. tuberculosis*

**Figure 2.**
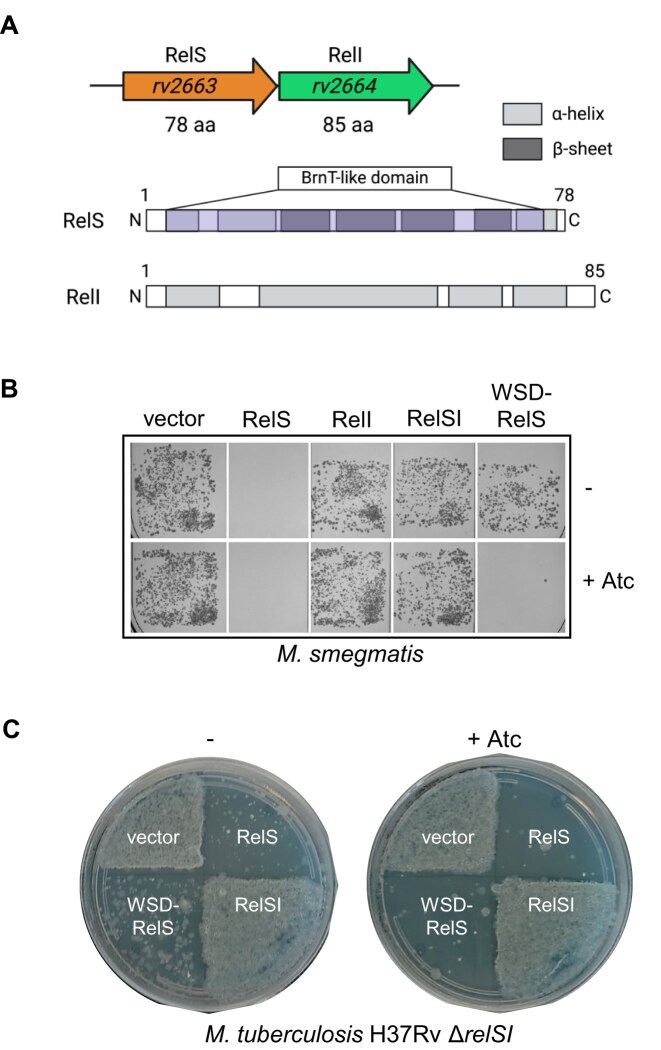
Rv2663–2664 (RelS–RelI) is a *bona fide* TA system in *M. tuberculosis*. (**A**) Schematic representation of *rv2663−2664* (*relS–relI*) of *M. tuberculosis*. The toxin gene is shown in light orange and the antitoxin gene in light green. The amino acid length of each protein product is given as a subscript, and the locus tag is shown within the gene schematic representation. Secondary structure elements are shown. (**B**) RelS toxicity is neutralized by RelI in *M. smegmatis. Mycobacterium smegmatis* transformed with pGMC-vector, -RelS, -RelI, -RelS–RelI, or RelS downstream of a weak Shine–Dalgarno sequence (-WSD-RelS) were plated on LB agar plates supplemented with or without Atc inducer at 100 ng ml^−1^. Plates were incubated 3 days at 37°C. Data are representative of at least three independent experiments. (**C**) RelS toxicity is neutralized by RelI in *M. tuberculosis* Δ*rv2663−2664* mutant strain. *Mycobacterium tuberculosis* H37Rv *Δrv2663–2664* transformed with pGMC-vector, -RelS, -RelS–RelI, or -WSD-RelS were plated on 7H11 agar plates supplemented with 10% OADC with or without Atc inducer at 200 ng ml^−1^. Plates were incubated for 3 weeks at 37°C. Data are representative of at least two independent experiments.

Rv2663–2664 likely combines a putative helix-turn-helix (HTH) DNA-binding protein (Rv2664, now named RelI) with a putative toxin (Rv2663, now named RelS) that has low sequence similarity to the Rel-like BrnT toxin of *Brucella abortus* [21]. HHpred search with RelI protein sequence as a query has not revealed any specific similarity with known antitoxins, but it identified a reliable (probability 99%) but partial similarity with ClpC2 protein within an HTH domain region (Supplementary Fig. S2A). ClpC2 is a self-repressor and a molecular sponge involved sequestrating an antitubercular drug, cyclomarin A [55]. The PSI-BLAST search (*E*-value = 1e^−5^) against a database of complete genomes identified 124 non-redundant proteins, which were used to build an alignment and reconstruct a phylogenetic tree (Supplementary Fig. S2B; Datasheet 1). All RelI homologs except two proteins are from Actinomycetes and can be assigned to two mostly monophyletic groups: (i) short proteins, most of which are encoded next to RelS toxins; (ii) proteins with fusions to either (*i*) the DUF3887 domain belonging to SnoaL/NTF2 superfamily that includes various enzymes as well as inactivated proteins [56], or (*ii*) to the DUF2812 domain with no reliable sequence similarity to any known protein families; these fused proteins are typically encoded by stand-alone genes. Thus, it appears that at least some of RelI homologs represent distinct DNA-binding antitoxins recently emerged from ClpC2. Our data demonstrate that RelS (78 amino acids) is acutely toxic in *M. smegmatis* (Fig. 1B) and that toxicity is inhibited when RelI (85 amino acids) is co-expressed either from the *rv2663–rv2664* operonic context or *in trans* from a different plasmid (Fig. 2A and B; Supplementary Fig. S3A). This indicates that RelS–RelI indeed performs as a *bona fide* TA system. Noticeably, we did not obtain any viable clones following transformation of *M. smegmatis* with pGMC-RelS, even in the absence of inducer (Fig. 1B), highlighting its highly toxic nature but also preventing further *in vivo* analysis of the toxin. In order to bypass this problem, we introduced a weak Shine–Dalgarno (SD) sequence in front of *rv2663* in the pGMC vector, as performed previously with the MenT3 toxin [30]. In this case, we successfully obtained viable clones in the absence of inducer (Fig. 2B). Note that this construct was used throughout this work for the *in vivo* characterization of RelS. In order to test whether RelS–RelI functions as a TA system in its native host, we first deleted the *rv2663–rv2664* operon in *M. tuberculosis* H37Rv to avoid possible toxin inhibition by the endogenous antitoxin, and then transformed the mutant with either RelS or RelS–RelI expressed from pGMC plasmids. The results from Fig. 2C show that RelS is indeed toxic in *M. tuberculosis* and that its toxicity is abolished in the presence of RelI. Together these data demonstrate that RelS–RelI (Rv2663-Rv2664) also functions as a *bona fide* TA system in *M. tuberculosis*.

### Structure of the RelS–RelI TA complex reveals a novel mode of RNase toxin inhibition

**Figure 3.**
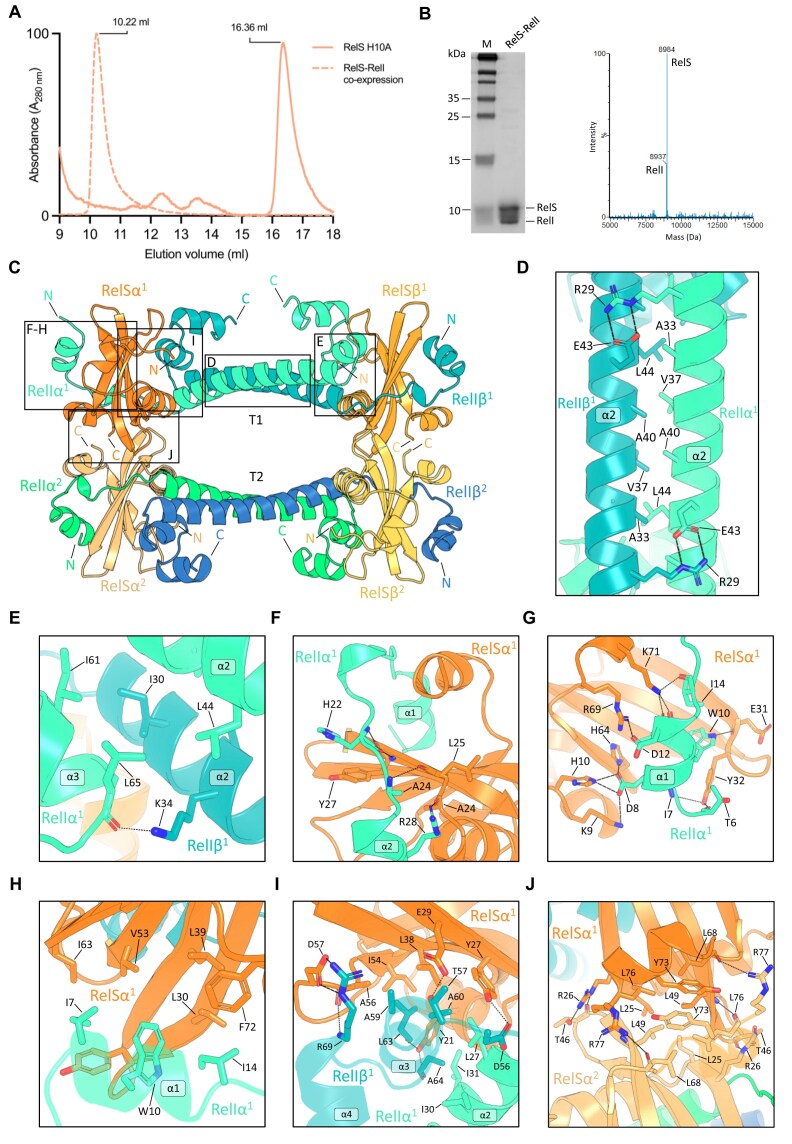
Structure of the RelS–RelI complex. (**A**) Overlay of SEC elution profiles corresponding to purified RelS H10A and RelS(WT)-RelI expression samples reveals co-expression of RelS and RelI results in the formation of a stable toxin–antitoxin complex. Purified protein samples were applied to a Superdex™ 75 increase 10/300 GL SEC column and eluted with 1.2 column volumes of analytical SEC buffer (20 mM Tris, pH 7.9, 150 mM NaCl). Chromatograms were exported, normalized between 0 and 100, and cropped to the appropriate scale. (**B**) SDS–PAGE and positive electrospray time-of-flight mass spectrometry (Es^+^-ToF MS) confirm the presence of both RelS and RelI within the final purified co-expression sample. (**C**) X-ray crystallographic structure of the [RelS_2_:RelI_2_]_2_ heterooctamer shown as a cartoon, colored by chain. N- and C-termini are indicated. (**D**–**J**) Close-up views of the corresponding boxed regions in panel (C), rotated to show key interacting residues within each interface. Residues are shown as sticks with atoms colored red for oxygen and blue for nitrogen. Interaction types are denoted by lines: short dashed line = hydrogen bond; long dashed line = salt bridge.

Although RelS–RelI functions as a TA system *in vivo*, the mechanism by which RelI inhibits RelS remained unknown. In order to address this, we first expressed RelS either in the absence or presence of RelI in *E. coli*. Akin to when expressed in *M. smegmatis* (Fig. 2B), RelS was too toxic to express alone in *E. coli*, whereas co-expression of RelS and RelI abolished cellular toxicity. To circumvent the inherent toxicity of RelS when expressed alone, we generated an alanine substitution mutant of a conserved histidine residue (H10A) previously shown to be important for BrnT toxicity [21], which when expressed alone in *E. coli* resulted in a non-toxic phenotype, allowing for sufficiently high yields of the toxin to be purified. We subsequently analyzed lone RelS H10A and co-expressed RelS WT-RelI samples by SEC using an analytical Superdex^TM^ 75 increase 10/300 GL column, which revealed that co-expression of RelS–RelI resulted in a 6.14 ml reduction in elution volume compared to RelS H10A expressed alone (Fig. 3A). Peak chromatographic fractions of the RelS–RelI co-expression sample were then analyzed by SDS–PAGE and positive electrospray time-of-flight mass spectrometry (Es^+^-ToF MS), which confirmed the presence of both RelS and RelI within the major SEC elution peak for this sample (Fig. 3B), supporting the resolved species to be a stable RelS:RelI TA complex. Having already calibrated this column prior to analysis using known molecular weight calibrants (GE Healthcare), we generated AlphaFold-predictive models of monomeric RelS H10A and RelS:RelI (assessing all possible stoichiometries), then performed correlation analyses to predict the stoichiometries of respective samples. Correlation of observed molecular weight (*M*_w_) and Stokes radius (*R*_st_) values for RelS H10A against values calculated using the monomeric RelS H10A AlphaFold model returned ratios of 0.82 and 0.90, respectively (Supplementary Fig. S3B), suggesting that RelS H10A exists as a monomer in solution. For the RelS–RelI co-expression sample, correlation analyses provided the highest-scoring ratios when the heterooctameric RelS:RelI AlphaFold model was used as a reference, yielding *M*_w_ and *R*_st_ values of 0.92 and 1.19, respectively (Supplementary Fig. S3B).

Having captured the RelS:RelI complex by SEC, we performed crystallization trials and subsequently solved the X-ray crystallographic structure of the RelS:RelI complex to a resolution of 1.70 Å (PDB 9SDC; Fig. 3C, Table 1), confirming the complex to be that of a heterooctamer. The final structure comprises two RelS_2_:RelI_2_ tetramers in the crystallographic asymmetric unit (denoted T1 and T2), which dimerize to form a stable heterooctamer with a 4:4 T:A ratio owing to primary interfaces between respective RelS toxins (RelS:RelS). PISA analysis [57] of the RelS:RelI crystal structure reveals that all calculated interfaces are almost identical within and between either tetramer, which comprise two RelS monomers (denoted RelSα^1^ and RelSβ^1^ for T1 and RelSα^2^ and RelSβ^2^ for T2), both of which adopt the expected RNase T1-like (or RelE-like, SCOP) fold, and two antiparallel RelI antitoxin protomers (denoted RelIα^1^ and RelIβ^1^ for T1, and RelIα^2^ and RelSI^2^ for T2). Sequence-independent structural alignment (superposition) of respective toxin protomers within either tetramer returned root mean square deviation (RMSD) values of 0.140 Å (T1; across 490 atoms [including all backbone and side chain atoms]) and 0.135 Å (T2; across 480 atoms), whilst superposition of respective antitoxins returned RMSD values of 0.232 Å (T1; across 471 atoms) and 0.254 Å (T2; across 449 atoms). Similarly, superposition of either tetramer returned an RMSD of 0.185 Å (across 1940 atoms), indicating near-identical topologies. For simplicity, interface analysis will focus solely on RelSα^1^, RelIα^1^, and RelIβ^1^ from T1 (and RelSα^2^ from T2 for RelS:RelS interfaces).

Within each heterotetramer, the RelI antitoxins dimerize to form a longitudinal scaffold via a widespread network of van der Waals interactions between recurrent hydrophobic amino acids scattered along respective α2 helices (Fig. 3D; calculated interface area = 1152.9 Å^2^). These interactions are further supported by salt bridges between R29 and E43, and hydrogen bonds between K34 and the backbone carbonyl oxygen of L65, the latter of which resides on the RelI α3 helix and partakes in van der Waals interactions with I30 on the α2 helix of the adjacent antitoxin (Fig. 3E). The N-terminal α1 helix of each antitoxin is connected to the elongated central α2 helix via a short 12-aa flexible loop that contacts the uppermost RelS β1 strand. Interactions consist primarily of hydrogen bonds (Fig. 3F), though π–π stacking and van der Waals interactions also ensure that the RelIα^1^ α1 helix arches across the extended β-sheet of the corresponding RelSα^1^ molecule (RelSα^1^:RelIα^1^). Specifically, RelIα^1^ α1 helix residues D8 and D12 form salt bridges with RelSα^1^ K9, H10, H64, and R69 (Fig. 3G); this interface is supported by hydrophobic van der Waals interactions and an extensive network of hydrogen bonds (Fig. 3H), which help lock the RelIα^1^ α1 helix in place. These interactions comprise one-third of the total interface surface (calculated interface area = 1055.4 Å^2^; total interface area = 3217.9 Å^2^), highlighting their importance in stable complex formation.

Several interactions were also observed between RelSα^1^ and the auxiliary RelIβ^1^ antitoxin, though these contributed to only 13% of the total interface area (calculated interface area = 422.2 Å^2^). Akin to the RelSα^1^:RelIα^1^ interface, the RelIβ^1^ α3 helix is anchored to the RelSα^1^ β-sheet by van der Waals contacts formed with amino acids located both on the anterior face of the toxin and on the α2 helix of the adjacent RelIα^1^ protomer, thus forming a second hydrophobic pocket (Fig. 3I). RelSα^1^ L38, I54, and A56 interact with RelIβ^1^ A59 and L63, whilst RelIα^1^ L27, I30, and I31 contact RelIβ^1^ residues A60 and A64. Hydrogen bonds were also observed between RelSα^1^ amino acids Y27 and E29 and RelIβ^1^ loop residues D56 and T57, and between RelSα^1^ Y21 and the main-chain oxygen atoms of RelIβ^1^ V63 and A64, both of which reside at the base of the α3 helix. This interface also houses two salt bridges between RelSα^1^ D57 and RelIβ^1^ R69. Similarly, RelSα^1^:RelSα^2^ interactions responsible for bridging respective tetramers account for only 18% of the total interface (calculated interface area = 587.4 Å^2^). A third hydrophobic pocket was observed within the buried area encased between interacting RelSα protomers comprising aliphatic amino acids L25, L49, L68, and L76 (Fig. 3J). The guanidino side-chains of R26 and R77 form hydrogen bonds with T46 and L68 on the opposite protomer (and vice versa), whilst the main-chain amide nitrogen of L49 hydrogen bonds with the main-chain carbonyl oxygen of L76. Finally, Y73 π-π stacks with the equivalent residue on the adjacent RelS protomer.

Based on the RelS:RelI crystal structure, we generated a series of RelI truncation mutants designed to abolish key interactions between RelS and RelI helices α1 (Δ2–24), α2 (Δ26–54), and α3/α4 (Δ57–84; Supplementary Fig. S3C), and subsequently tested their ability to neutralize RelS toxicity in *M. smegmatis*. As we had observed in *M. tuberculosis*, induction of RelS alone resulted in a severely toxic phenotype *in vivo*, whilst expression of RelI WT efficiently blocked cellular toxicity (Supplementary Fig. S3D). In contrast, RelI Δ2–24 was unable to block RelS toxicity, as demonstrated by a toxic phenotype comparable to that of RelS when expressed alone, whilst Δ26–54 and Δ57–84 truncation mutants could only partially rescue *M. smegmatis* growth (Supplementary Fig. S3D). Next, we co-expressed RelS and RelI Δ2–24 in *E. coli* (which is more tolerant to RelS) and performed *in vitro* pull-downs using His-SUMO-tagged RelS as bait to determine whether the inability of RelI Δ2–24 to neutralize RelS *in vivo* was due to an inability to bind. SDS–PAGE analysis of soluble protein content following clarification of cell lysate identified a faint, low-molecular-weight band around the expected size of the RelI Δ2–24 mutant (Supplementary Fig. S3E; “Sol”), indicating that deletion of the RelI N-terminus had not detrimentally affected protein folding or stability. However, no RelI Δ2–24 could be observed in the final purified RelS sample following cleavage of the His-SUMO tag (Supplementary Fig. S3E; “Final”), which is in sharp contrast to when RelI WT was co-expressed with His-SUMO-RelS, wherein a strong interaction was observed between respective proteins (Fig. 3B). The absence of such an interaction between His-SUMO-RelS and RelI Δ2–24 indicates that residues 2–24 are essential for complex formation and antitoxicity. Together with observation that steady-state levels of overexpressed His-tagged RelI WT and RelI Δ2–24 are comparable (Supplementary Fig. S3F), these data support the claim that RelI likely blocks RelS toxicity through steric occlusion of the highly electropositive toxin active site (Supplementary Fig. S3G).

### RelS inhibits translation

**Figure 4.**
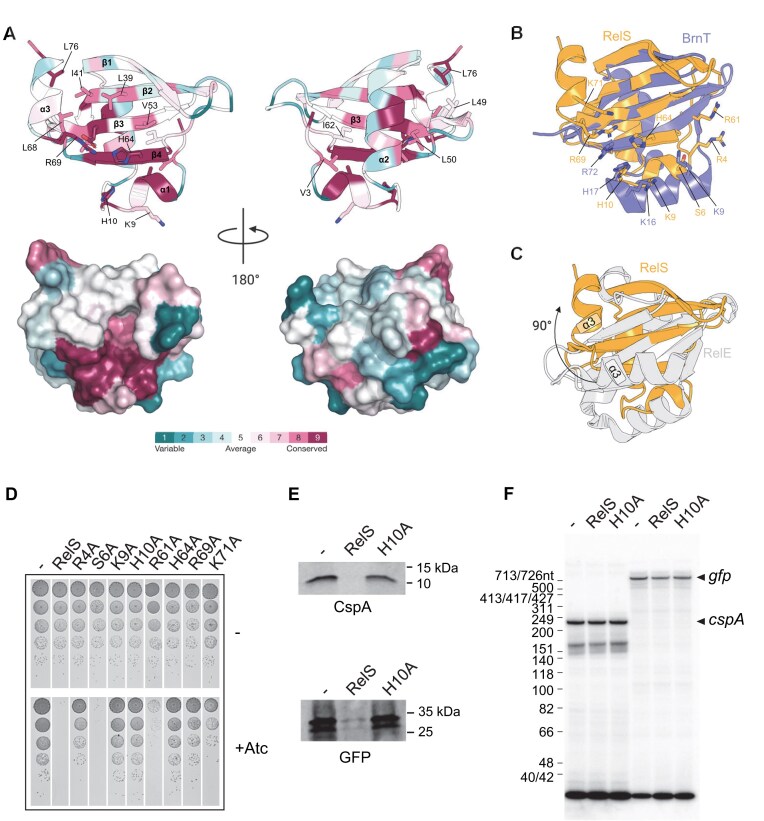
RelS inhibits translation. (**A**) ConSURF analysis of *M. tuberculosis* RelS shown from the front (*left*) and rear (*right*). Conserved residues are shown in magenta, whereas variable residues are colored teal, as per provided scale. (**B, C**) Superposition of RelS (colored light orange) onto BrnT from *B. abortus* (B; PDB 3U97), RMSD 2.66 Å across 214 atoms; and RelE from *E. coli* (C; PDB 4FXI), RMSD 5.36 Å across 236 atoms. Putative catalytic residues from BrnT that are conserved in RelS are shown as sticks with atoms colored red for oxygen and blue for nitrogen, with RelS residues of interest also shown for reference. (**D**) Toxicity of RelS derivatives in *M. smegmatis. Mycobacterium smegmatis* transformed with pGMC-vector, -WSD-RelS, or its mutant derivatives (alanine substitution of residues R4, S6, K9, H10, R61, H64, R69, or K71) were serially diluted and spotted on LB agar plates with or without Atc inducer at 100 ng ml^−1^. Plates were incubated for 3 days at 37°C. Data are representative of three independent experiments. (**E**) RelS inhibits protein synthesis *in vitro. Mycobacterium smegmatis in vitro* translation reactions assessing the synthesis of CspA or GFP protein in the absence (–) or presence of RelS toxins (5 µM). Reactions were performed for 2 h at 37°C, and newly synthesized CspA and GFP were labeled with [^35^S]-methionine. Samples were separated on SDS–PAGE and visualized using a phosphorimager. Data are representative of at least three independent experiments. (**F**) RelS does not cleave mRNA *in vitro*. RNAs were extracted from *M. smegmatis in vitro* translation reactions in the absence (–) or presence of RelS toxins (5 µM). Primer extension was carried out at 48°C for 1 h with [^32^P]-labeled *cspA* or *gfp* extension primer. The obtained labeled cDNAs were separated on denaturing urea-polyacrylamide gel and revealed by autoradiography. Arrows show uncleaved *cspA* (126 nt) and *gfp* (565 nt). Data are representative of two to three independent experiments.

Having characterized the mechanism of RelS inhibition by RelI, we sought to elucidate the toxic mode of action for RelS. The structure of RelS is similar to other Rel superfamily toxins and consists of a central 4-stranded β-sheet core encased between two α-helices (α2 and α3). ConSURF [58] analysis of amino acid conservation revealed that several residues within sheets β2–4 are highly conserved (Fig. 4A). Within the β2 and β3 sheets, L39, I41, L50, and V53 form hydrophobic interactions with the α2 and α3 helices, helping to support positioning on either face of the toxin. β4 amino acid H64, involved in binding of RelI, is highly conserved, whereas α3 and β1 amino acids show high variability, likely a result of evolutionary divergence from related RelE homologues to enable binding of specific targets. The structure of RelS also revealed structural similarities with the BrnT toxin from *B. abortus* [21]. Superposition of RelS with BrnT (PDB 3U97) returned an RMSD of 2.66 Å (across 214 atoms), indicating an overall degree of shared homology between respective toxin structures (Fig. 4B), with high conservation of residues such as K9, H10, and R69 in RelS that share respective positions with residues K16, H17, and R72 shown to contribute to BrnT toxicity (Fig. 4B). Unlike RelS, BrnT lacks the C-terminal α3 helix that vertically spans sheets β2–4. However, this helix has been resolved in the structure of *E. coli* RelE that was obtained in the absence of cognate antitoxin RelB (PDB 4FXI), which, when aligned with that of RelS from the RelS:RelI complex, gives an RMSD of 5.36 Å (across 236 atoms) (Fig. 4C). In isolated *E. coli* RelE, the α3 helix is rotated 90° clockwise relative to the corresponding helix of RelS and instead runs horizontally across the β2 and β3 sheets of the toxin. Binding of the cognate RelB antitoxin induces a conformational change to the toxin that results in displacement of the α3 helix. We therefore propose that binding of RelI may well induce a similar conformational change to the RelS toxin that leads to displacement of the C-terminal α3 helix, potentially from a conserved hydrophobic patch comprised of L39 and V53 in RelS (Fig. 4A–C). Antitoxicity, therefore, potentially functions through a combination of conformational changes to RelS and steric occlusion of RelS catalytic residues.

Among the eight putative catalytic site residues tested, R4A, R69A, and K71A substitutions resulted in a slight reduction in cellular toxicity, while K9A, H10A, and H64A severely affected RelS toxicity (Fig. 4D). Both K9 and H10 were shown to be important for BrnT toxicity [21], indicating that RelS and BrnT may share a similar catalytic mechanism. When tested both K9A and H10A substitutions also abolished RelS toxicity when expressed in the *M. tuberculosis* Δ*rv2663–2664* mutant strain (Supplementary Fig. S4A). Previous work showed that BrnT could function as a ribosome-independent RNase capable of cleaving *lacZ* model mRNA *in vitro* [21]. In order to investigate RelS activity *in vitro*, we purified both RelS WT and the inactive RelS H10A mutant and tested their RNase activity using both purified *cspA* and *gfp* as model mRNA targets. In sharp contrast to BrnT, RelS did not cleave mRNA *in vitro* (Supplementary Fig. S4B). Therefore, we next investigated whether RelS inhibits translation using an *M. smegmatis*-based translation assay in the presence of purified *cspA* or *gfp* mRNA. In this case, we found that RelS WT, but not RelS H10A, could inhibit synthesis of both CspA and GFP (Fig. 4E). We next performed primer extension experiments on the translation reactions obtained in the presence or absence of the toxin [41] and show that both *cspA* and *gfp* mRNA were not detectably cleaved by RelS (Fig. 4F). Note that as a control, we show that *cspA* was efficiently cleaved by the ribosome-dependent toxins HigB1 and RelE3/YoeB under the same conditions (Supplementary Fig. S4C). Together these data show that RelS inhibits translation but does not cleave mRNA.

### RelS specifically targets the 16S ribosomal RNA

**Figure 5.**
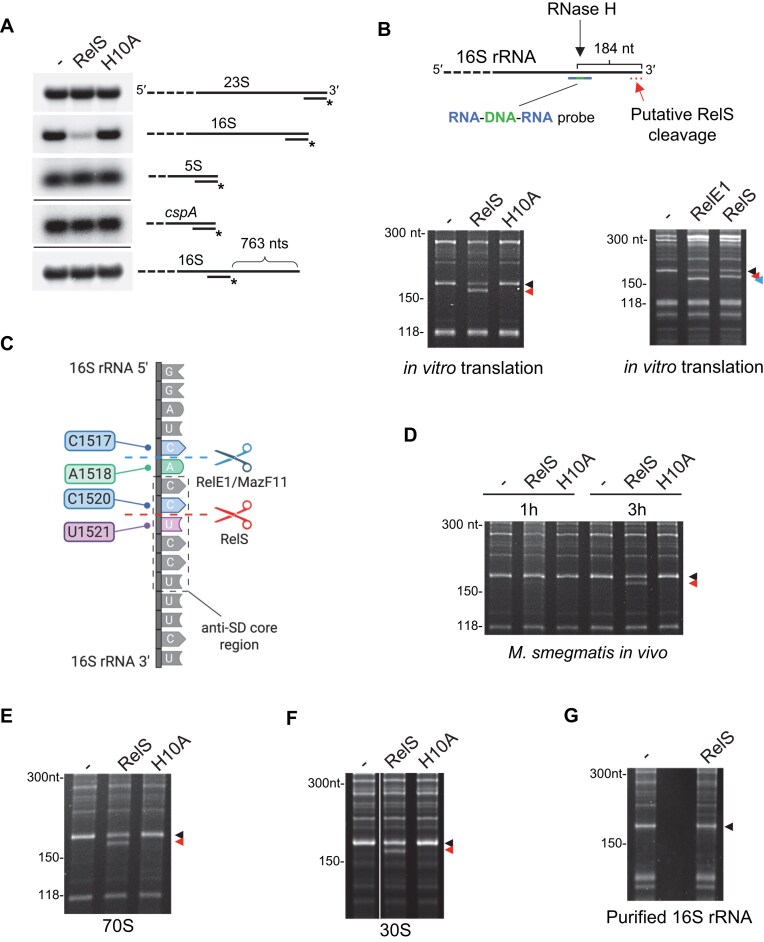
RelS cleavage of 16S rRNA within the anti-SD does not require ongoing translation. (**A**) Northern blot analysis of RNAs extracted from *M. smegmatis in vitro* translation reactions, using [^32^P]-labeled 23S, 16S, and 5S rRNA, *cspA* mRNA 3′ end probes, and a probe located at the middle part of the 16S rRNA. RNAs were extracted in the absence (−) or presence of toxins (5 µM), separated on 1.2% agarose gels, and transferred overnight to Amersham Hybond-N+ membranes. Membranes were then hybridized with [^32^P]-labeled oligonucleotide probes at 42°C overnight. The radioactive membranes were exposed to phosphorimager screens, and the signals were measured using a Typhoon Imager. Data are representative of at least three independent experiments. (**B**) RelS WT, but not RelS H10A, cleaves the 16S rRNA 3′ end. A DNA–RNA–DNA hybrid probe (blue and green color) positioned at 184 nt from the 3′ end of the 16S rRNA was used to promote RNase H cleavage and generate 16S rRNA fragments that can be separated and sequenced. RNAs were extracted from *M. smegmatis in vitro* translation reactions in the absence (–) or presence of toxins (5 µM) and subjected to a RNase H treatment. The obtained RNAs were separated on 6% denaturing urea–polyacrylamide gels, stained by SYBR^TM^ Safe, and visualized by UV epi-illumination. The black triangle shows the expected uncleaved 16S rRNA 3′ end, the red triangle shows the RelS-cleaved fragment, and the blue triangle shows the known RelE1-cleaved fragment. Data are representative of at least three independent experiments for RelS cleavage and two for RelS/RelE1 comparison. (**C**) RelS toxin cleavage occurs between C1520 and U1521 within the anti-SD region of 16S rRNA. (**D**) RelS cleaves the 16S rRNA *in vivo*. RelS or RelS H10A was expressed from a pGMC-based vector for 1 or 3 h in *M. smegmatis* in the presence of Atc inducer. Total RNA from two independent experiments was extracted, subjected to a RNase H treatment, separated, and stained as described in (B). Purified *M. smegmatis* 70S ribosome (**E**), 30S subunit (**F**), or 16 rRNA from RNA extracts (**G**) were incubated with 5 µM of RelS WT or RelS H10A, or without toxin at 37°C for 2 h. Following the incubations, RNAs were extracted and subjected to RNase H treatment. The obtained RNAs were separated and stained as described in Fig. 5. The black triangle shows the 16S rRNA 3′ end RNA fragment of uncleaved RNA (~180 nt), and the red triangle shows the RelS-cleaved RNA fragment. The data for panels (A) and (B) are representative of three independent experiments. The data for panel (C) are representative of two independent experiments.

We then asked whether RelS could target rRNA, as observed for other toxins like VapC or MazF [59, 60], and more recently for the RelE1 toxin of *M. tuberculosis* [42]. RNA from *M. smegmatis*-based *in vitro* translation experiments obtained in the presence of RelS WT or RelS H10A were extracted, and northern blots were performed using probes located at the 3′ ends of *M. smegmatis* 16S and 23S rRNAs (Fig. 5A). Remarkably, a significant decrease in the 16S rRNA level was found in the presence of RelS WT, while RelS H10A had no effect. No RelS cleavage was detected in 23S and 5S rRNA, or in *cspA* and *gfp* mRNA. These data indicate that RelS likely inhibits translation by cleaving the 16S rRNA. Noticeably, when northern blots were performed with a probe located in the middle part of 16S rRNA, we could not detect a decrease in the size and intensity of the 16S rRNA band, indicating that rRNA cleavage by RelS occurs toward the 3′ end region of the 16S rRNA (Fig. 5A).

We next applied an RNase H-based method in order to detect short differences in size between RNA fragments. In this case, we used an RNA/DNA/RNA hybrid probe that specifically recognizes the 16S rRNA of *M. smegmatis* close to its 3′ end, producing a fragment of ~180 nt long after RNase H treatment for the mature 16S rRNA in the absence of toxin or in the presence of the inactive RelS H10A toxin (Fig. 5B). In sharp contrast, a shorter 16S rRNA fragment was observed in the presence of RelS WT, indicating that RelS indeed cleaves before the 3′ end of the 16S rRNA (Fig. 5B). Remarkably, cleavage of the 16S rRNA by RelS produced a longer fragment than the one observed for RelE1 (Fig. 5B), indicating that RelS cleaves downstream of the recently identified RelE1 cleavage site.

Further 3′-OH sequencing of the 16S rRNA fragments precisely identified RelS cleavage between nucleotides U1521 and C1520 of the 16S rRNA, within the anti-SD sequence and 3 nt downstream of the RelE1 and MazF11 cleavage site (Fig. 5C and Supplementary Fig. S4D). Accordingly, RelS produced the same cleavage *in vivo* after 1 and 3 h (i.e. one doubling time) of expression in *M. smegmatis* (Fig. 5D). Together, these data indicate that 16S rRNA is the main target of RelS and that cleavage by the toxin leads to the formation of ribosomes deprived of a functional anti-SD region, likely reflecting its acute toxicity.

Although RelS cleaves the 16S rRNA, it is not clear whether such activity would require active translation. In order to test this hypothesis, we purified both *M. smegmatis* 70S and 30S ribosomal fractions and incubated them separately with purified RelS WT or RelS H10A, then extracted RNA and performed an RNase H treatment to detect 16S rRNA cleavage. The data presented in Fig. 5E and F show that RelS WT, but not RelS H10A, cleaves the 16S rRNA from the purified 70S ribosome and 30S subunit, thus indicating that RelS does not need ongoing translation for catalysis. In addition, the fact that RelS WT could not cleave free purified 16S rRNA (Fig. 5G) strongly suggests that cleavage of the 16S rRNA 3′ end by RelS requires an assembled 30S ribosomal subunit.

### Structural models support the importance of catalytic RelS residues for RNase activity

**Figure 6.**
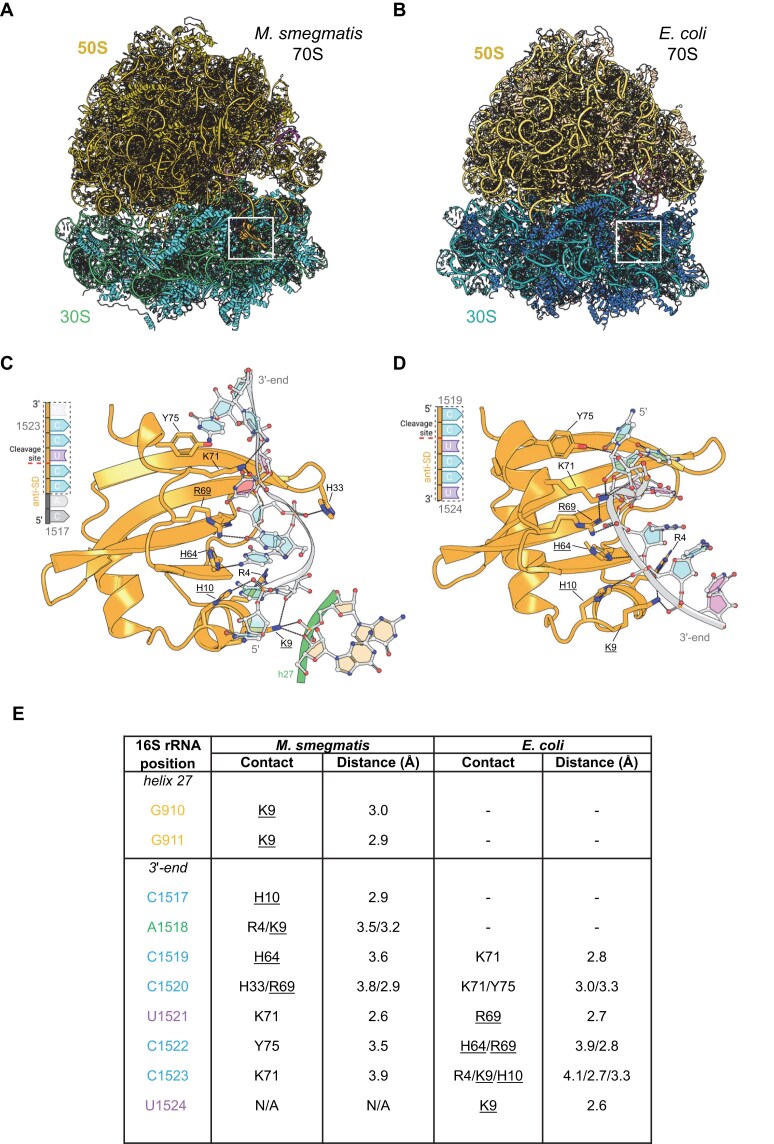
Molecular docking studies predict catalytic RelS residues are important for RNA recognition. Molecular docking of RelS to *M. smegmatis* (**A**) and *E. coli* (**B**) 16S rRNA. Docking models are structurally aligned against full 70S ribosomes colored by subunit, with RelS colored orange. The structures of RelS, *M. smegmatis* 16S rRNA, and *E. coli* 16S rRNA were extracted from the RelS:RelI crystal structure (PDB 9SDC) and the full *M. smegmatis* and *E. coli* ribosomes (PDB 8WI9 and 5AFI, respectively), then uploaded to HADDOCK for docking, selecting only the final 3′ 16S rRNA bases as active residues. Close-up views of the boxed regions in panels (A) and (B) showing RelS docked to *M. smegmatis* (**C**) and *E. coli* (**D**) 30S subunits. Predicted interacting residues are shown as sticks with atoms colored red for oxygen, blue for nitrogen, and orange for phosphorous. 16S rRNA nucleotides are shown as ball-and-stick cartoons colored gray, with rings colored by nucleotide (A = green; C = blue; G = yellow; U = violet). Corresponding nucleotide sequences and their respective orientations are shown to the left of either panel. The underlined residues were shown to be important for RelS toxicity in *M. smegmatis*. (**E**) Table summarising the residues of RelS predicted to interact with the 16S rRNA of *M. smegmatis* and *E. coli*.

In order to investigate whether RelS could bind to the 30S ribosomal subunit in the vicinity of the anti-SD region of the 16S rRNA, we utilized HADDOCK [61] to generate predictive models of RelS bound to the 30S subunit, using *M. smegmatis* and *E. coli* 16S rRNAs as templates for docking (PDB 8WI9 and 5AFI, respectively). It is worth noting that full 70S ribosomes were not used, as the computational jobs were too large. The resulting models show that RelS is indeed capable of binding to the 30S subunit of either species in close proximity to the 3′ end region of 16S rRNA (Fig. 6A and B), with either model predicting a network of protein–RNA interactions with nucleotides housed within the conserved anti-SD core (C1519–U1524).

RelS appears to form a larger number of contacts with the *M. smegmatis* 30S subunit than when docked to the corresponding subunit from *E. coli* (Fig. 6C and D). Specifically, conserved toxin residues K9 and H10 contact A1518 and C1517, respectively, both of which reside directly upstream of the anti-SD core, with K9 also predicted to interact with the phosphodiester backbone of the 16S rRNA within helix 27 (Fig. 6C). No such contacts are formed with the equivalent nucleotides in the *E. coli* 30S subunit (Fig. 6E), perhaps hinting at a structural mechanism for the comparably higher levels of cellular toxicity in mycobacteria. Toward the extreme 3′ end, the *M. smegmatis* 16S anti-SD is guided vertically across the RelS surface through a channel of positively charged residues encompassing R4, H33, H64, R69, and K71 (Fig. 6C), culminating with base-stacking interactions between Y75 and C1523. Of these amino acids, H64 and R69 are indispensable for RelS toxicity *in vivo*, with single alanine substitution of R4 and K71 also impacting toxicity (Fig. 4D).

Within the *E. coli* 30S subunit, the anti-SD extends across the RelS surface in the opposite direction as is observed for the *M. smegmatis* 16S anti-SD, suggestive of highly varied binding mechanisms to respective subunits (Supplementary Fig. S5A and B). Structural alignment of the RelS:16S rRNA model onto the full *E. coli* 30S subunit also suggests that binding of RelS would require displacement of the highly disordered N-terminus of ribosomal protein S21 to allow the toxin to occupy the small cavity encased between the 16S rRNA 3′ end and ribosomal proteins S7 and S11 (Supplementary Fig. S5B). Once again, this region is predicted to form contacts with RelS amino acids K9, H10, H64, and R69 (Fig. 6D), all of which are essential for RelS toxicity *in vivo* (Fig. 4D) and collectively form the primary interface with the RelI antitoxin in the resolved RelS:RelI crystal structure (Fig. 3G). The predicted role of each of these residues in contacting the anti-SD further supports the idea that binding of the antitoxin precludes RNA substrate recognition by these specific amino acids. Overall, our predictive docking models offer a structural rationale for the potential role of conserved residues contributing to RelS toxicity, whilst supporting binding of the toxin to the 30S subunit near the site of 16S rRNA cleavage (Fig. 5C).

## Discussion

In this work, we used a computational approach to predict previously unnoticed novel TA pairs (RelS–RelI) in the human pathogen *M. tuberculosis*. We studied the toxic mechanism of RelS and elucidated the mode of neutralization by RelI, showing that toxicity is efficiently blocked through steric hindrance of essential catalytic residues. We show that the RelS toxin, which belongs to the RelE superfamily and shares structural homology with the *B. abortus* BrnT toxin, is acutely toxic in *M. tuberculosis* and specifically targets the 30S ribosomal subunit, cleaving the exposed 3ʹ end region of the 16S rRNA between C1520 and U1521, within the anti-SD region, a position that is so far unique to RelS. Unlike the cognate antitoxins of other RelE superfamily toxins, the newly identified RelI antitoxin is not part of any known antitoxin superfamily, but instead belongs to a small family of HTH domain-containing proteins with partial similarity to ClpC2 that are specific for *Actinomycetes*. Previous CRISPRi screening revealed that *relI* lies within the list of most vulnerable genes in *M. tuberculosis* and that is likely essential for survival [62], thus suggesting that the antitoxin could represent a susceptible drug target to control growth of the pathogen via RelS activation.

Determination of the RelSI toxin–antitoxin complex by X-ray crystallography shows that it is structurally distinct from previously solved complexes of Rel-family toxins with their cognate antitoxins, which is in agreement with the absence of known structural homologues of RelI in the PDB (as identified through DALI) [63]. RelS inhibition by RelI is dependent on wrapping of the RelI N-terminal region across the surface of RelS, likely leading to displacement of the α3 helix and both deactivation of the active site and sequestration of RelS. Whilst such a mode of inhibition resembles the one observed for RelBE [42, 64], dimerization in RelB forms a coiled-coil region that ends in a C-terminal DNA-binding domain important for transcriptional regulation. Remarkably, whilst RelI dimerization also forms a short coiled-coil region, its C-terminus is made up of two shorter helices, which contribute to binding of the second partner RelS toxin protomer. This arrangement, wherein each RelS toxin makes two discrete RelI antitoxin interactions, showed very tight regulation requiring extensive mutagenesis to break. As RelI is unlike known antitoxins, it provides further evidence for the dynamic flexibility between TA partners, with multiple folds known to inhibit Rel/Par superfamily toxins [21, 65].

As RelI sequesters RelS directly, RelSI can be considered as a type II TA system. However, there is currently no evidence that RelSI would be able to bind DNA and perform the transcriptional regulation associated with type II TA [66]. Indeed, the structure shows that the putative HTH region of RelI forms a complex interaction network with RelS and so might not permit DNA binding (Fig. 3I). Neither examination of the complex electrostatic surface, nor attempts to model DNA-bound RelSI complexes using AlphaFold3 [67], produced any strong evidence for likely modes of DNA binding. Analysis of the upstream 1000 bp region also did not reveal likely *cis*-acting regulatory elements such as inverted repeats. RelSI is in the more rare gene order wherein the toxin is encoded before the antitoxin, as seen for *Proteus vulgaris* HigBA, *E. coli* MqsRA, and *B. abortus* BrnTA, all of which encode Rel-family toxins [21, 65, 68]. In the case of *mqsRA*, the full operon is transcriptionally repressed by the MqsA antitoxin, which is itself constitutively expressed from additional promoters located within the *mqsR* coding region [68]. Importantly, these antitoxin-specific promoters are not transcriptionally autoregulated by MqsA, ensuring higher transcriptional rates of antitoxin are achieved. To determine whether *relS* and *relI* are differentially transcribed, the *relS* nucleotide sequence was submitted to BPROM [69] in a bid to identify potential *relI*-specific promoters. However, no putative promoter elements were identified in the *relS* coding region, suggesting *relS–relI* are transcribed from a single promoter, which would correlate to equal rates of transcription. Thus, *M. tuberculosis* may rely on higher translational rates of RelI relative to RelS to achieve higher cytosolic levels of antitoxin, as has been reported for other Type II TA systems [70]. Future work should aim to characterize the modes of transcriptional and translational regulation for RelSI to elucidate how the relative abundancies of toxin and antitoxin are regulated, both under normal physiological conditions and in response to stress.

Targeting the 30S ribosomal subunit to inhibit bacterial growth is not restricted to RelS [71]. Yet, the anti-SD region of the 16S rRNA is only recently emerging as a hot spot for poisonous toxins, especially in *M. tuberculosis*, as shown for RelE1 and MazF11 toxins [42, 72]. Indeed, both unrelated toxins were shown to preferentially cleave the 3′ end of 16S rRNA 1 nt upstream of the anti-SD sequence between nucleotides C1517 and A1518, 3 nt upstream of the identified RelS cleavage site. Like RelS, RelE1 is also reliant on fully assembled 30S subunits for 16S rRNA cleavage, potentially reflecting similar binding mechanisms to the 30S ribosomal subunit [42]. In addition, for both toxins, the toxicity data support predictive models showing both accessibility of the 16S rRNA 3′ end region and complementary interactions between the toxins and 16S rRNA, which would facilitate such a toxic mechanism. The novel catalytic function shared by both Rel toxins indicates that these toxins are markedly different from ribosome-dependent mRNA interferase toxins such as HigB1, YoeB, and RelE (Feng *et al*., 2013; Mansour *et al*., 2022; Neubauer *et al*., 2009).

Accumulation of 30S subunits deprived of their anti-SD region may lead to a severe drop in translation initiation and to the subsequent inhibition of bacterial growth and eventually cell death. In contrast with mRNA cleavage during translation and the production of stalled ribosomes that can potentially be rescued by tmRNA [73], cleavage of the anti-SD region on free 30S subunits (as well as on 70S ribosomes) suggests an irreversible collapse of translation, which is in agreement with the acute toxicity of both RelS and RelE1 in mycobacteria. Prior investigations in *E. coli* showed that starvation stress induces the formation of a substantial pool of free ribosomal subunits that are subsequently targeted for efficient degradation by endogenous RNases [74, 75]. Remarkably, such processes initiate when the exonuclease RNase PH first removes ~20 nt from the 3ʹ end of the 16S rRNA, which then triggers the rRNA degradation cascade mediated by other RNases, including RNase E, RNase II, and RNase R [74, 76]. Although this has not been investigated in *M. tuberculosis*, we hypothesize that initial cleavage by RelS, RelE1, or MazF11 *in vivo* could trigger a similar process. Although *relS* is among the genes that are highly expressed under long-term starvation conditions in *M. tuberculosis* [77, 78], the only known phenotype associated with its mutation so far is a reduced overall fitness when exposed to the aminoglycoside antibiotic streptomycin [79]. How cleavage of the anti-SD region by RelS could somehow reduce the impact of streptomycin and confer a survival advantage in response to antibiotic challenge in *M. tuberculosis* is currently unknown. While both streptomycin and RelS target the 16S rRNA, streptomycin specifically interacts at the decoding site to impair the ribosome’s ability to accurately decode mRNA [80], which is not in the vicinity of the identified RelS activity (Fig. 6). Although very hypothetical, diminution of the pool of active ribosomes in the cell by stress-activated RelS might reduce the number of viable streptomycin targets and thus provide a fitness advantage. More work is warranted to address such a possibility.

The unique anti-SD-targeting catalytic activity of RelE1 and RelS toxins differs from other Rel-family toxins in that neither toxin seem to act at the ribosomal A-site. Superposition of RelE1 onto each of the ribosome-bound structures of these toxins had provided hints as to why RelE1 does not bind to the A-site [42], demonstrating a lack of structurally equivalent RNA-interacting residues in RelE1 otherwise present in RelE, HigB1, and YoeB. Structural alignments show that each of these toxins is substantially larger than RelS, which would likely occupy a far smaller volume within the ribosomal A-site relative to each of the three *bona fide* mRNase toxins (Supplementary Fig. S5C–F). Conserved RelE residues responsible for contacting the decoding centre are missing in RelS, whilst the RelS α1 helix is much shorter than the equivalent helix in RelE and lies ∼7 Å displaced from h31. Similarly, the RelS α3 helix is rotated compared to the equivalent helices in both RelE and HigB1, which clashes with the resolved mRNAs in either structure. The same can be said of YoeB; of the known RNA-interacting basic residues, only one is structurally conserved in RelS. In all instances, the catalytic centres of each mRNase toxin lie far displaced from the putative RelS catalytic core. Therefore, despite the similar folds shared between Rel superfamily proteins, mRNase toxins are functionally distinct from RelS and RelE1. Accordingly, structural alignments of RelS against RelE1 show that putative catalytic residues align well (Supplementary Fig. S5F), supporting the divergent mode of action of these toxins from the canonical catalytic mechanism employed by RelE, HigB1, and YoeB. Yet, it remains to be investigated whether other Rel homologs from bacteria or archaea have a similar target as RelS and RelE1.

Collectively, these data provide further evidence that the 16S rRNA 3ʹ end is a previously overlooked hotspot for translational control. In addition, further mining of TA-rich species will likely continue to deliver novel biology with the potential to invigorate therapeutic applications.

## Supplementary Material

gkag571_Supplemental_Files

## Data Availability

The crystal structure of RelSI complex has been deposited in the Protein Data Bank under accession number 9SDC (https://doi.org/10.2210/pdb9sdc/pdb). Ribosomal rRNA 3′ seq generated in this project have been deposited at the European Nucleotide Archive database under accession code PRJEB110532. All other data needed to evaluate the conclusions in the paper are present in the manuscript, in the Supplementary File or Datasheet 1.
