# Supplementary material for: Ribosomal RNA cleavage by the previously unidentified RelS–RelI toxin–antitoxin system controls growth of Mycobacterium tuberculosis
Source: Nucleic Acids Res. 2026 Jun 22;54(12):gkag571. doi: 10.1093/nar/gkag571 (PMC13284704; doi:10.1093/nar/gkag571)
Supplement: gkag571_Supplemental_Files [file gkag571_supplemental_files.zip › RelSI_SUPPLEMENTARY FILES 05 05 26.pdf]

SUPPLEMENTARY FILES

Ribosomal RNA cleavage by the previously unidentified RelS-RelI toxin-antitoxin system controls growth of *Mycobacterium tuberculosis*

Xue Han, Tom J. Arrowsmith, Svetlana Karamycheva, Xibing Xu, Michèle Coddeville, Carine Pagès, Bertille Voisin, Claude Gutierrez, Olivier Neyrolles, Kira S. Makarova, Tim R. Blower, Pierre Genevaux

This file contains 5 Supplementary Figures and 2 Tables.

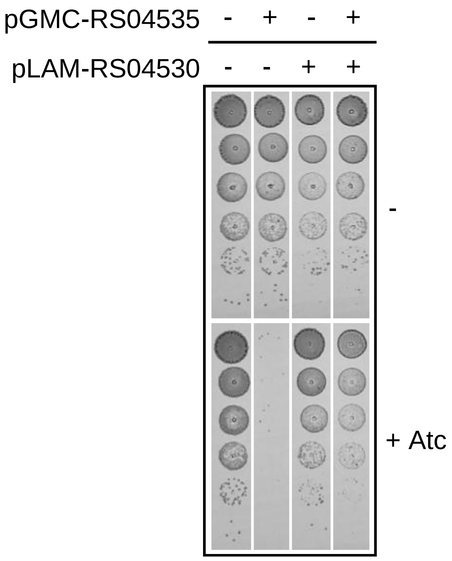

Supplementary Fig. 1: **AWB99_RS04530 and AWB99_RS04535 can function as a TA system *in vivo*.** *M. smegmatis* was co-transformed with pGMC-vector or pGMC-RS04535, and pLAM-vector or pLAM- RS04530, serially diluted and spotted on LB agar plates with or without Atc inducer at 100 ng·ml^{-1}. Plates were incubated 3 days at 37 °C. Data are representative of two independent experiments.

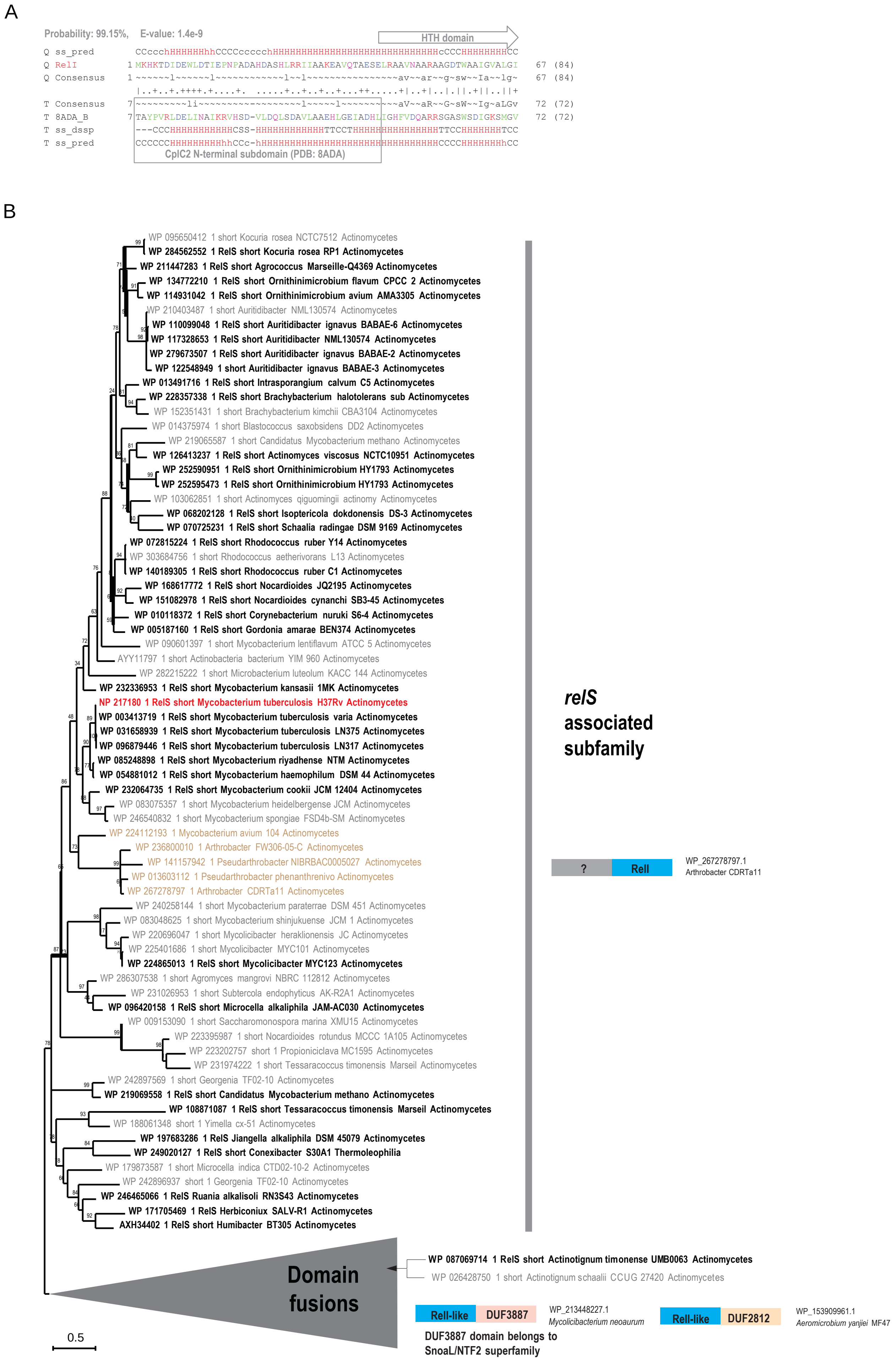
A
Probability: 99.15%, E-value: 1.4e-9
HTH domain
Q ss_pred CCccchHHHHHHhhCCCCccccchHHHHHHHHHHHHHHHHHHHHHHHHHHHHHHHcCCCHHHHHHHHCC
Q RelI 1 MKHKTDIDEWLDTIEPNPADAHDASHLRRIIAAKEAVQTAESELRAAVNAARAAGDTWAAIGVALGI 67 (84)
Q Consensus 1 ~~~~~~l~~~~~~l~~~~~~~~~~~~~~l~~l~~~~~~~~~~~~~~~~av~~ar~~g~sw~~Ia~~lg~ 67 (84)
T Consensus 7 ~~~~~~~~~~li~~~~~~~~~~~~~~~~~~l~~~~~l~~~~~~~~~~aV~~aR~~G~sW~~Ig~aLGv 72 (72)
T 8ADA_B 7 TAYPVRLDELINAIKRVHSD-VLDQLSDAVLAAEHLGEIADHLIGHFVDQARRSGASWSDIGKSMGV 72 (72)
T ss_dssp ---CCCHHHHHHHHHCSS-HHHHHHHHHHHHTTCCTHHHHHHHHHHHHHHHHHTTCCHHHHHHHTCC
T ss_pred CCCCCCHHHHHHHHHhhCCCc-hHHHHHHHHHHHHHHHHHHHHHHHHHHHHHHHHCCCCHHHHHHHhCC
CplC2 N-terminal subdomain (PDB: 8ADA)
B
WP 095650412 1 short Kocuria rosea NCTC7512 Actinomycetes
WP 284562552 1 RelS short Kocuria rosea RP1 Actinomycetes
WP 211447283 1 RelS short Agrococcus Marseille-Q4369 Actinomycetes
WP 134772210 1 RelS short Ornithinimicrobium flavum CPCC 2 Actinomycetes
WP 114931042 1 RelS short Ornithinimicrobium avium AMA3305 Actinomycetes
WP 210403487 1 short Auritidibacter NML130574 Actinomycetes
WP 110099048 1 RelS short Auritidibacter ignavus BABAE-6 Actinomycetes
WP 117328653 1 RelS short Auritidibacter NML130574 Actinomycetes
WP 279673507 1 RelS short Auritidibacter ignavus BABAE-2 Actinomycetes
WP 122548949 1 short Auritidibacter ignavus BABAE-3 Actinomycetes
WP 013491716 1 RelS short Intrasporangium calvum C5 Actinomycetes
WP 228357338 1 RelS short Brachybacterium halotolerans sub Actinomycetes
WP 152351431 1 short Brachybacterium kimchii CBA3104 Actinomycetes
WP 014375974 1 short Blastococcus saxobsidens DD2 Actinomycetes
WP 219065587 1 short Candidatus Mycobacterium methano Actinomycetes
WP 126413237 1 RelS short Actinomyces viscosus NCTC10951 Actinomycetes
WP 252590951 1 RelS short Ornithinimicrobium HY1793 Actinomycetes
WP 252595473 1 RelS short Ornithinimicrobium HY1793 Actinomycetes
WP 103062851 1 short Actinomyces qiguomingii actinomy Actinomycetes
WP 068202128 1 RelS short Isoptericola dokdonensis DS-3 Actinomycetes
WP 070725231 1 RelS short Schaalia radingae DSM 9169 Actinomycetes
WP 072815224 1 RelS short Rhodococcus ruber Y14 Actinomycetes
WP 303684756 1 short Rhodococcus aetherivorans L13 Actinomycetes
WP 140189305 1 RelS short Rhodococcus ruber C1 Actinomycetes
WP 168617772 1 RelS short Nocardioides JQ2195 Actinomycetes
WP 151082978 1 RelS short Nocardioides cynanchi SB3-45 Actinomycetes
WP 010118372 1 RelS short Corynebacterium nuruki S6-4 Actinomycetes
WP 005187160 1 RelS short Gordonia amarae BEN374 Actinomycetes
WP 090601397 1 short Mycobacterium lentiflavum ATCC 5 Actinomycetes
AYY11797 1 short Actinobacteria bacterium YIM 960 Actinomycetes
WP 282215222 1 short Microbacterium luteolum KACC 144 Actinomycetes
WP 232336953 1 RelS short Mycobacterium kansasii 1MK Actinomycetes
NP 217180 1 RelS short Mycobacterium tuberculosis H37Rv Actinomycetes
WP 003413719 1 RelS short Mycobacterium tuberculosis varia Actinomycetes
WP 031658939 1 RelS short Mycobacterium tuberculosis LN375 Actinomycetes
WP 096879446 1 RelS short Mycobacterium tuberculosis LN317 Actinomycetes
WP 085248898 1 RelS short Mycobacterium riyadhense NTM Actinomycetes
WP 054881012 1 RelS short Mycobacterium haemophilum DSM 44 Actinomycetes
WP 232064735 1 RelS short Mycobacterium cookii JCM 12404 Actinomycetes
WP 083075357 1 short Mycobacterium heidelbergense JCM Actinomycetes
WP 246540832 1 short Mycobacterium spongiae FSD4b-SM Actinomycetes
WP 224112193 1 Mycobacterium avium 104 Actinomycetes
WP 236800010 1 Arthrobacter FW306-05-C Actinomycetes
WP 141157942 1 Pseudarthrobacter NIBRBAC0005027 Actinomycetes
WP 013603112 1 Pseudarthrobacter phenanthrenivo Actinomycetes
WP 267278797 1 Arthrobacter CDRTa11 Actinomycetes
WP 240258144 1 short Mycobacterium paraterrae DSM 451 Actinomycetes
WP 083048625 1 short Mycobacterium shinjukuense JCM 1 Actinomycetes
WP 220696047 1 short Mycolicibacter heraklionensis JC Actinomycetes
WP 225401686 1 short Mycolicibacter MYC101 Actinomycetes
WP 224865013 1 RelS short Mycolicibacter MYC123 Actinomycetes
WP 286307538 1 short Agromyces mangrovi NBRC 112812 Actinomycetes
WP 231026953 1 short Subtercola endophyticus AK-R2A1 Actinomycetes
WP 096420158 1 RelS short Microcella alkaliphila JAM-AC030 Actinomycetes
WP 009153090 1 short Saccharomonospora marina XMU15 Actinomycetes
WP 223395987 1 short Nocardioides rotundus MCCC 1A105 Actinomycetes
WP 223202757 short 1 Propioniciclava MC1595 Actinomycetes
WP 231974222 1 short Tessaracoccus timonensis Marseil Actinomycetes
WP 242897569 1 short Georgenia TF02-10 Actinomycetes
WP 219069558 1 RelS short Candidatus Mycobacterium methano Actinomycetes
WP 108871087 1 RelS short Tessaracoccus timonensis Marseil Actinomycetes
WP 188061348 short 1 Yimella cx-51 Actinomycetes
WP 197683286 1 RelS Jiangella alkaliphila DSM 45079 Actinomycetes
WP 249020127 1 RelS short Conexibacter S30A1 Thermoleophilia
WP 179873587 1 short Microcella indica CTD02-10-2 Actinomycetes
WP 242896937 short 1 Georgenia TF02-10 Actinomycetes
WP 246465066 1 RelS Ruania alkalisoli RN3S43 Actinomycetes
WP 171705469 1 RelS Herbiconiux SALV-R1 Actinomycetes
AXH34402 1 RelS short Humibacter BT305 Actinomycetes
relS associated subfamily
? RelI
WP_267278797.1
Arthrobacter CDRTa11
Domain fusions
WP 087069714 1 RelS short Actinotignum timonense UMB0063 Actinomycetes
WP 026428750 1 short Actinotignum schaalii CCUG 27420 Actinomycetes
RelI-like DUF3887
WP_213448227.1
Mycolicibacterium neoaurum
RelI-like DUF2812
WP_153909961.1
Aeromicrobium yanjiei MF47
DUF3887 domain belongs to SnoaL/NTF2 superfamily
0.5

Supplementary Fig. 2: **Phylogenetic and sequence analysis RelI homologs.** (**A**) HHpred output showing similarity between RelI and ClpC2 (PDB:) protein families covering the region N-terminal region and HTH domain of ClpC2**.** (**B**) Homologs of RelI were identified in the in-house database of 47545 completely sequences genomes downloaded from Genbank in November 2023 using PSI-BLAST program with Evalue=1e-5. Sequences were aligned using MUSCLE5 program and phylogenetic tree was built for DNA-binding domain only (82 positions) using FastTree program as described in the Methods section. Sequence analysis revealed that many RelI homologs are fused to other domain, so those which are not fused were classified as short (indicated in the tree). Presence of adjacent relS gene is also indicted in the leafs description along with the protein accession, organism name and bacterial lineage. The tree schematics shows two large clades mostly consistent with presence or absence of fusions with other domains. The proteins from the clade with mostly short proteins (black and gray) are often encoded in the operon with relS genes (shown by bold in the tree) and represent subfamily of true RelI orthologs, with a possible exception of a small branch colored brown, where HTH domain is fused to an unknown N-terminal domain (schematics on the right) and a small branch with short RelI-like proteins in the second clade (which could be an artifact because the proteins are shorter than others). Two typical fusions of RelI-like HTH domain found in the proteins within the collapsed branch are also shown on the right of the branch.

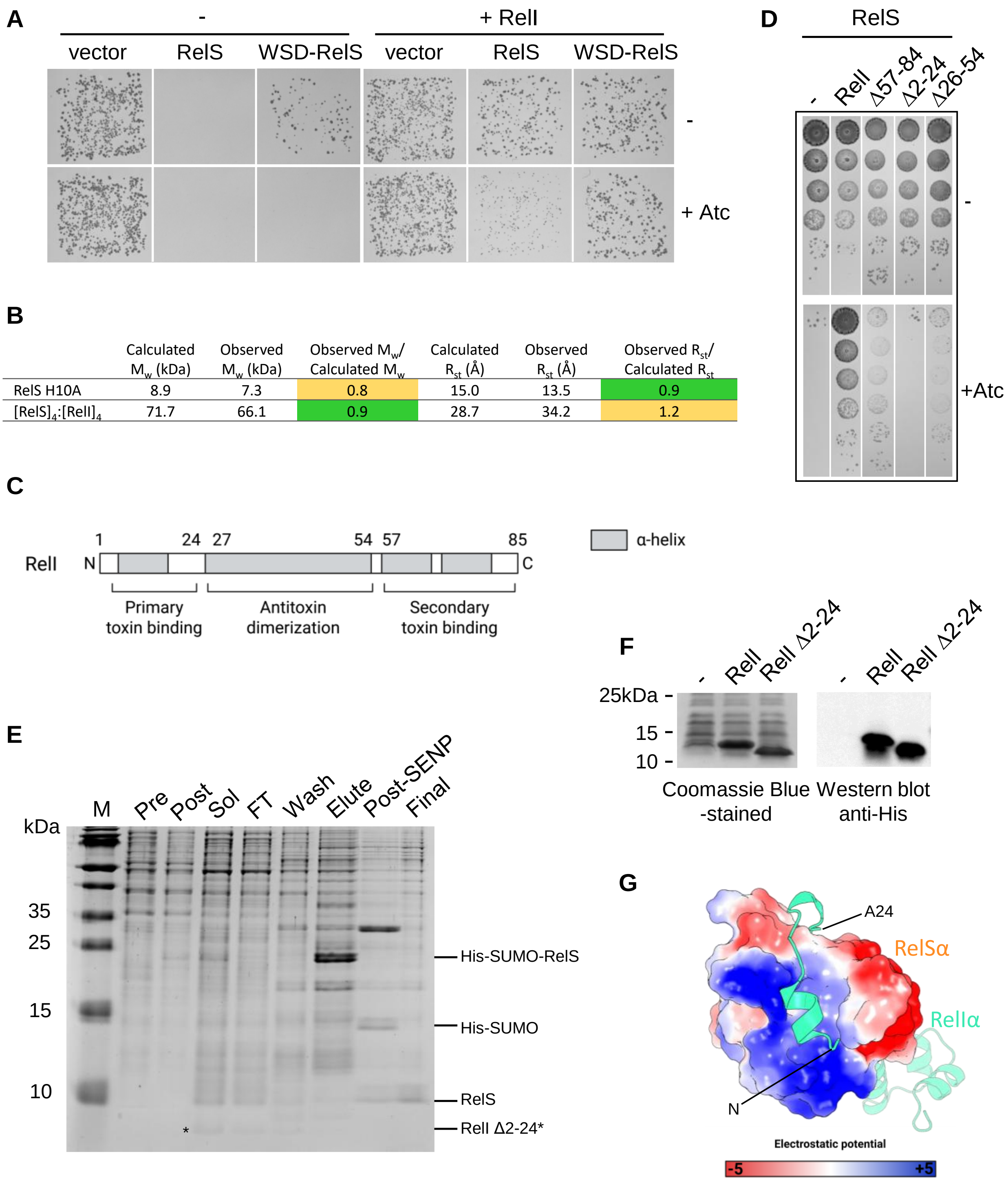

	Calculated M_w (kDa)	Observed M_w (kDa)	Observed M_w/ Calculated M_w	Calculated R_{st} (Å)	Observed R_{st} (Å)	Observed R_{st}/ Calculated R_{st}
RelS H10A	8.9	7.3	0.8	15.0	13.5	0.9
$[RelS]_4$:$[RelI]_4$	71.7	66.1	0.9	28.7	34.2	1.2

Supplementary Fig. 3: RelI inhibits the toxicity of RelS in *M. smegmatis* and requires the N-terminal 24 residues. (**A**) *M. smegmatis* co-transformed with pGMC-vector, RelS, WSD-RelS and pLAM12-vector or pLAM-RelI were plated on LB agar plates supplemented with or without Atc inducer at 100 ng·ml^{-1}. Plates were incubated 3 days at 37 °C. Data are representative of two independent experiments. (**B**) Correlation of observed molecular weight (M_w) and Stokes radius (R_{st}) values for lone RelS H10A and RelS-RelI co-expression samples against calculated values obtained from AlphaFold-predictive models of monomeric RelS H10A and the RelS:RelI heterooctamer. (**C**)

Schematic illustrating core RelI domains and their roles in toxin binding (2-24, 57-84) and antitoxin dimerization (26-54). (**D**) *M. smegmatis* co-transformed with pGMC-WSD-RelS and pLAM12 vector, RelI, RelI (Δ2-24), RelI (Δ26-54) or RelI (Δ57-84) were serially diluted and spotted on LB agar plates with or without Atc inducer at 100 ng·ml^{-1}. Plates were incubated for 3 days at 37 °C. Data are representative of two independent experiments. (**E**) *E. coli* DH5α co-transformed with pTRB686 (His-SUMO-RelS) and pTRB743 (RelIΔ2-24) were supplemented with 0.2% L-ara and 1 mM IPTG at mid-log phase for induction of TA expression and grown at 37 °C overnight. Cultures were lysed and contents clarified prior to affinity chromatography purification and SDS-PAGE analysis. No pull-down of RelI (Δ2-24) could be detected following co-expression with RelS, confirming N-terminal residues are essential for antitoxic activity. (**F**) Steady state levels of His-tagged RelI and RelI Δ2-24 in pET15b expressed in BL21 AI strain in the presence of 0.2% arabinose inducer overnight at 22°C. The coomassie Blue stained SDS-PAGE of whole cell extracts (left) and the corresponding western blot using anti-His antibody (right) are shown. (**G**) Surface electrostatics of RelSα bound to RelIα from the RelS:RelI crystal structure. Surface electrostatics depict electrostatic potential from −5 $kBTe^{-1}$ (red) to +5 $kBTe^{-1}$ (blue), where e is the electron, T is temperature and kB is the Boltzmann constant.

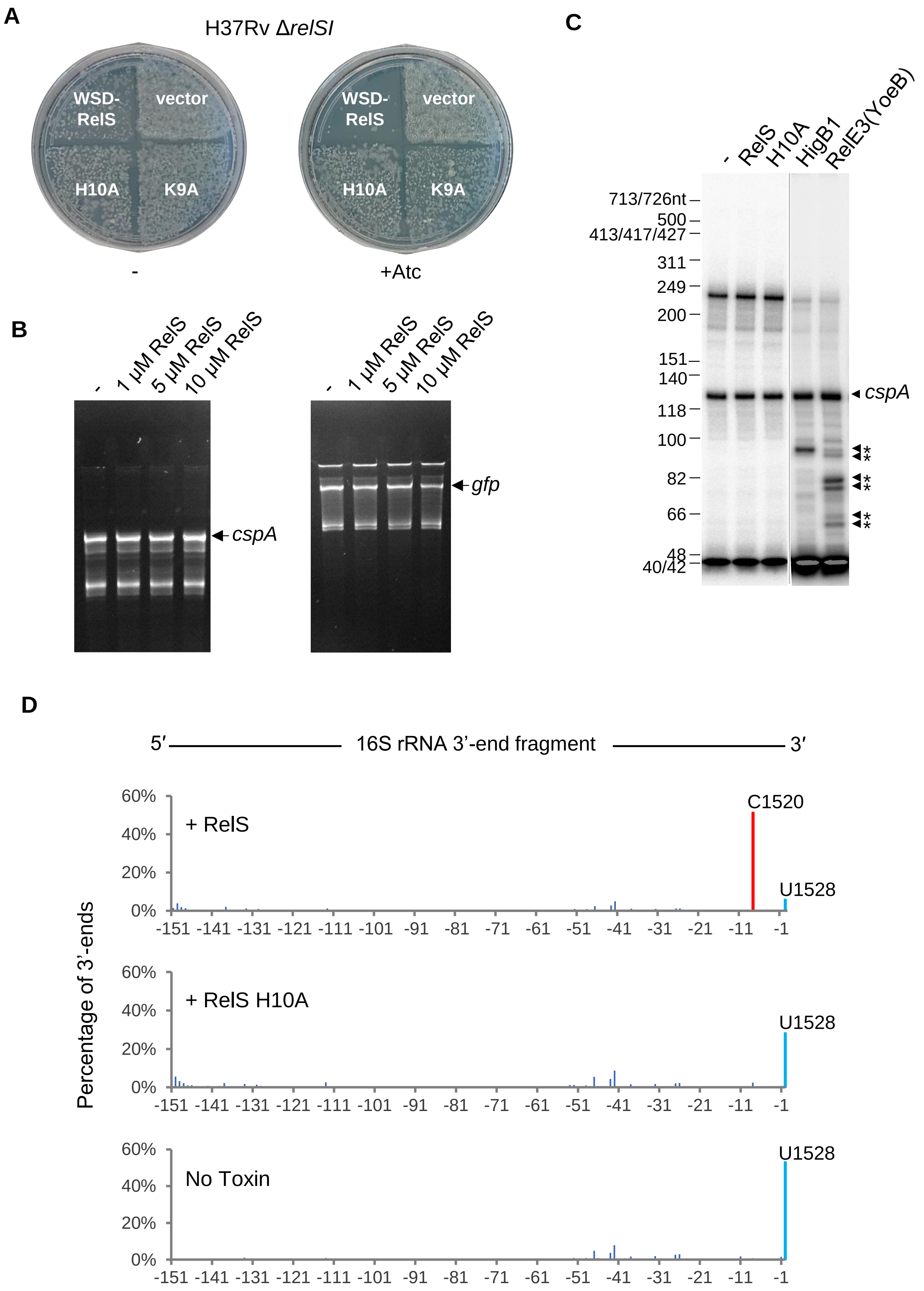

Supplementary Fig. 4: RelS is toxic in *M. tuberculosis*. (**A**) Toxicity of RelS derivatives in *M. tuberculosis* H37Rv *ΔrelSI*. *M. tuberculosis* Δ*relSI* transformed with pGMC-vector, WSD-RelS or its mutant derivatives (alanine substitution of residue K9 or H10 within RelS) were plated on 7H11 agar

plates supplemented with 10% OADC with or without Atc inducer at 200 ng·ml^{-1}. Plates were incubated 3 weeks at 37 °C. Data are representative of at least two independent experiments. (**B**) RelS does not cleave purified *cspA* or *gfp* mRNA. *In vitro* incubation in the absence (-) or presence of increasing concentrations of RelS toxin (1, 5 and 10 µM). The incubations were performed for 1 h at 37 °C and then products were separated on denaturing urea-polyacrylamide gel. RNAs were stained with SYBR^{TM} Safe diluted 10,000-fold in 1× TBE buffer and visualized by UV epi-illumination. Data are representative of two independent experiments. (**C**) HigB1 and RelE3/YoeB toxins, but not RelS cleave mRNA *in vitro M. smegmatis* translation reaction. RNAs were extracted from *M. smegmatis in vitro* translation reactions in the presence or in the absence of toxin. Primer extension was then performed at 48°C for 1h with [^{32}P]-labeled *cspA* primer. The obtained labeled cDNAs were separated on denaturing urea-polyacrylamide gel and revealed by autoradiography. Arrows show the uncleaved (*cspA*, 126 nt) and cleaved (*, 95 nt) *cspA*. RelS cleavage was performed more than three times and HigB1/RelE3(YoeB) twice independently. (**D**) RelS cleavage on 16S rRNA 3′-end. Mapping was performed using 1 µg of total RNA from *M. smegmatis* cell-free transcription-translation systems with or without RelS wild-type or RelS H10A substitution. The rRNA-seq libraries were then prepared and sequenced as described in the materials and methods section. Percentage of cleavages identified (y axis) at a specific position (x axis) within the 16S rRNA 3′-ends is shown. Representative data from two independent experiments are presented. Original data are shown in Datasheet 1.

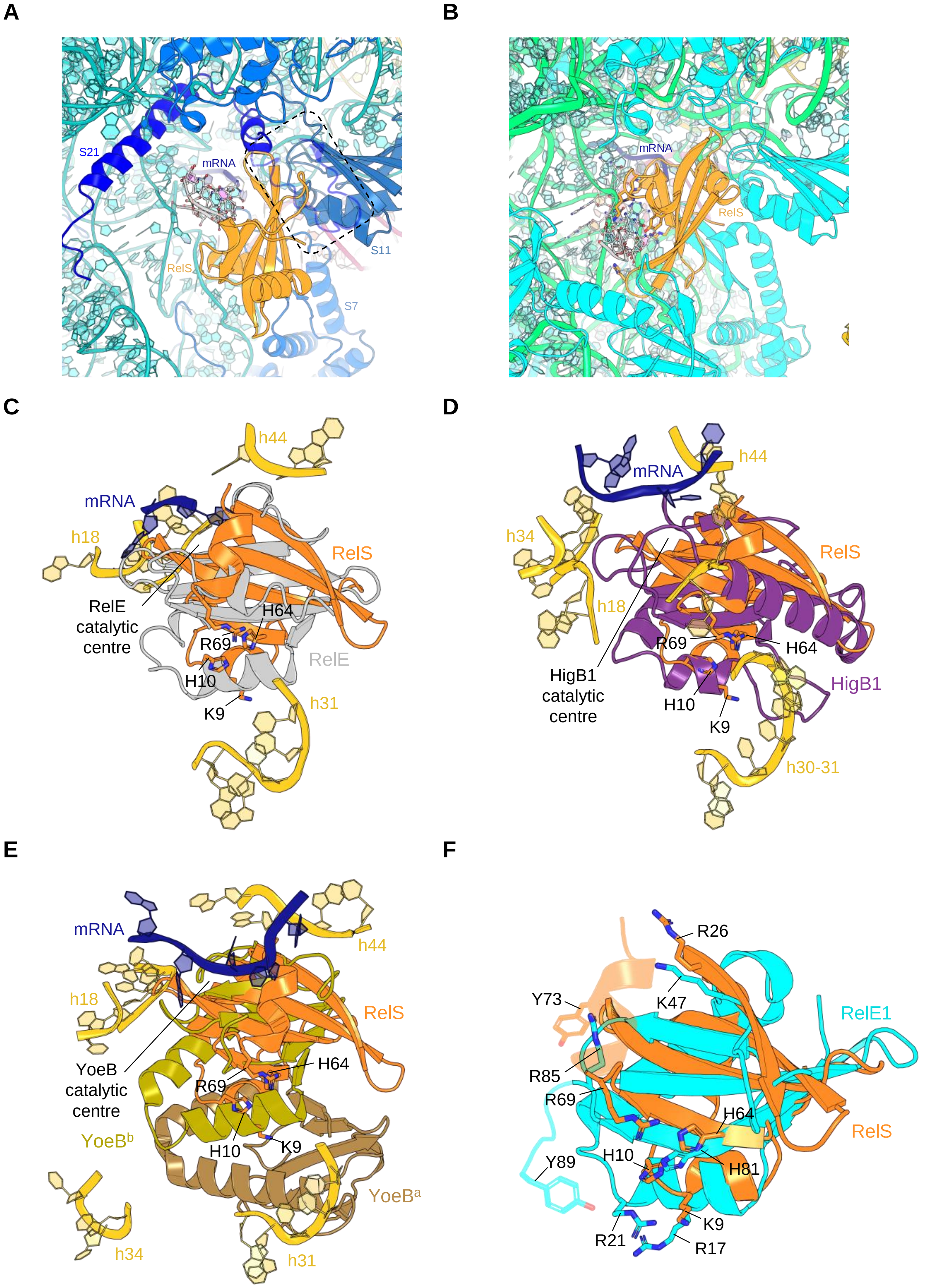

Supplementary Fig. 5: RelS molecular docking and superposition of RelS against related toxins bound to the ribosomal A site. (A, B) Close-up views of RelS bound to *E. coli* (**A**) and *M.*

smegmatis (**B**) 16S rRNA following molecular docking and superposition of models onto full 30S subunits. RelS clashes with the disordered N-terminal region of ribosomal protein S21 (shaded semi-transparent in the dashed box), suggesting that conformational changes would be required to accommodate the toxin within the cavity formed between ribosomal proteins S7, S11, and the 16S anti-SD region. No steric clashes were observed following superposition of the RelS:16S docking model onto the full *M. smegmatis* 30S subunit. **(C-F)** Sequence-independent structural alignment of RelS (orange) onto (**C**) *E. coli* RelE (PDB 4V7J, RMSD 6.025 Å across 295 atoms, gray), (**D**) *M. tuberculosis* HigB1 TAC (PDB 7NBU, RMSD 3.867 Å across 205 atoms; purple), and (**E**) *E. coli* YoeB (PDB 4V8X, RMSD 2.674 Å across 149 atoms, olive), all bound to the A-site of the 30S subunit. 16S rRNA are coloured gold and labels correspond to the helices on which interacting bases reside. (**F**) Sequence-independent structural alignment of RelS (orange) onto *M. tuberculosis* RelE1 (PDB 9G12, RMSD 4.565 Å across 286 atoms, cyan). Structurally conserved residues of interest are shown as sticks with atoms coloured red for oxygen and blue for nitrogen.

Supplementary Table S1: List of primers used in this work

Name	Sequence (5′-3′)
**Construction of *M. tuberculosis* chromosomal mutant**	
Rv2663-Am-Fw:	GCTCGCCGAGCACCTCGG
Rv2663-Zeo-Am-Rv:	CAGTCGATCCACGTGGAGAGGATGCCGTGCTTGCGGGC
Rv2664-Zeo-Av-Fw:	CCACTGAGCGTCAGACCCACGTGCTCGGGCCACACAGCACAGCGAG
Rv2664-Av-Rv:	GCGGCAACGCACCCGCTG
Zeo-Dir:	CTCCACGTGGATCGACTGCCAGGC
Zeo-Rev:	GAGCACGTGGGTCTGACGCTCAGTGG
Rv2664-int-Fw:	CTCGGCATCACCCGCCAGG
Rv2663-int-Rev:	CGCCGTGGTATTCCAGTTCGAC
**Toxin and antitoxin cloning in pGMC**	
Vector pGMC Fw	CAACTTTATTATACATAGTTGATAATTC
Vector pGMC Rv	GGGCAGCCTGTCTTCCTC
pGMC-Rv2663_Fw:	GAAGACAGGCTGCCCATGGAGGTGAGGGCT
pGMC-Rv2663_Rv:	TGTATAATAAAGTTGTCACCTCAGGTAGTC
pGMC-Rv2664_Fw:	GAAGACAGGCTGCCCATGAAGCACAAGACC
pGMC-Rv2664_Rv:	TGTATAATAAAGTTGTTAGGGGCTCGCTGT
pGMC-AWB99_RS04530_Fw:	GAAGACAGGCTGCCCATGCAGCTGAACAGG
pGMC-AWB99_RS04530_Rv:	TGTATAATAAAGTTGTCAGGACCTCCTTGCC
pGMC-AWB99_RS04535_Fw:	GAAGACAGGCTGCCCATGGCGGGGCAACGC
pGMC-AWB99_RS04535_Rv:	TGTATAATAAAGTTGCTACCCCCGAGCGGC
pGMC-Mycch_5153_Fw:	GAAGACAGGCTGCCCATGAGTCACGTCGAG
pGMC-Mycch_5153_Rv:	TGTATAATAAAGTTGTCATCGCCCCGCTTT
pGMC-Mycch_5154_Fw:	GAAGACAGGCTGCCCATGATGGAACGCGGC
pGMC-Mycch_5154_Rv:	TGTATAATAAAGTTGCTAGCGGTTGGTCGG
pGMC-BST38_RS17710_Fw:	GAAGACAGGCTGCCCATGACCGTCATAGTG
pGMC-BST38_RS17710_Rv:	TGTATAATAAAGTTGTCAGCGCCGAAATCG
pGMC-BST38_RS29380_Fw:	GAAGACAGGCTGCCCATGGCTTCGCCACAA
pGMC-BST38_RS29380_Rv:	TGTATAATAAAGTTGTCAGCGCACCTTCTC
pGMC-BLW81_RS13130_Fw:	GAAGACAGGCTGCCCATGACAGCAGCAACG
pGMC-BLW81_RS13130_Rv:	TGTATAATAAAGTTGTCACGCCGAGGCTGC
pGMC-BLW81_RS13135_Fw:	GAAGACAGGCTGCCCATGAGAAGCGCGGGA
pGMC-BLW81_RS13135_Rv:	TGTATAATAAAGTTGTCACCACAGCGTGGC
pGMC-AFA91_01820_Fw:	GAAGACAGGCTGCCCATGTATGCCGTCGCA
pGMC-AFA91_01820_Rv:	TGTATAATAAAGTTGTCAGCTCAGCGGCGC

pGMC-AFA91_01825_Fw:	GAAGACAGGCTGCCCATGGACCGGCTCACC
pGMC-AFA91_01825_Rv:	TGTATAATAAAGTTGTCAGAGGCGCTGACG
pGMC-Mycch_5672_Fw:	GAAGACAGGCTGCCCATGACCACCATCAAG
pGMC-Mycch_5672_Rv:	TGTATAATAAAGTTGTCAACCGCGTTCGGT
pGMC-Mycch_5673_Fw:	GAAGACAGGCTGCCCATGATCCATACCACC
pGMC-Mycch_5673_Rv:	TGTATAATAAAGTTGTCAGGTGTTGTCGGA
pGMC-Rv2663_weakSD_Fw:	CTTGTACAAAGTGGACGAAGACAGGCTGCC
pGMC-Rv2663_weakSD_Rv:	GGCAGCCTGTCTTCGTCCACTTTGTACAAG
Rv2663_R4A_Fw:	TGCCCATGGAGGTGGAGGCTAGCGCCCGCAAG
Rv2663_R4A_Rv:	CTTGCGGGCGCTAGCCTCCACCTCCATGGGCA
Rv2663_S6A_Fw:	ATGGAGGTGAGGGCTGCCGCCCGCAAGCACGGCA
Rv2663_S6A_Rv:	TGCCGTGCTTGCGGGCGGCAGCCCTCACCTCCAT
Rv2663_K9A_Fw:	AGGGCTAGCGCCCGCGCGCACGGCATCAACGA
Rv2663_K9A_Rv:	TCGTTGATGCCGTGCGCGCGGGCGCTAGCCCT
Rv2663_H10A_Fw:	GCTAGCGCCCGCAAGGCCGGCATCAACGACGA
Rv2663_H10A_Rv:	TCGTCGTTGATGCCGGCCTTGCGGGCGCTAGC
Rv2663_L25A_Fw:	CATACCGCAACGCGGCGCGCTACGTCGAACT
Rv2663_L25A_Rv:	AGTTCGACGTAGCGCGCCGCGTTGCGGTATG
Rv2663_Y27A_Fw:	ACCGCAACGCGCTGCGCGCCGTCGAACTGGAATAC
Rv2663_Y27A_Rv:	GTATTCCAGTTCGACGGCGCGCAGCGCGTTGCGGT
Rv2663_Y32A_Fw:	TACGTCGAACTGGAAGCCCACGGCGAAGTTCA
Rv2663_Y32A_Rv:	TGAACTTCGCCGTGGGCTTCCAGTTCGACGTA
Rv2663_H33A_Fw:	GTCGAACTGGAATACGCCGGCGAAGTTCAAC
Rv2663_H33A_Rv:	GTTGAACTTCGCCGGCGTATTCCAGTTCGAC
Rv2663_T46A_Fw:	ATCGGCCCCGACCAAGCCGGGCGCCTTTTAG
Rv2663_T46A_Rv:	CTAAAAGGCGCCCGGCTTGGTCGGGGCCGAT
Rv2663_R61A_Fw:	AGCAGACGAACCACCCGCGATTATCCACGCCAAC
Rv2663_R61A_Rv:	GTTGGCGTGGATAATCGCGGGTGGTTCGTCTGCT
Rv2663_H64A_Fw:	ACCACCCCGGATTATCGCCGCCAACGTACTAC
Rv2663_H64A_Rv:	GTAGTACGTTGGCGGCGATAATCCGGGGTGGT
Rv2663_R69A_Fw:	CACGCCAACGTACTAGCCCCGAAGTTCTACGA
Rv2663_R69A_Rv:	TCGTAGAACTTCGGGGCTAGTACGTTGGCGTG
Rv2663_K71A_Fw:	AACGTACTACGCCCGGCGTTCTACGACTACCTG
Rv2663_ K71A_Rv:	CAGGTAGTCGTAGAACGCCGGGCGTAGTACGTT
pGMC-63_64_Fw:	GAAGACAGGCTGCCCATGGAGGTGAGGGCT
pGMC-63_64_Rv:	TGTATAATAAAGTTGTTAGGGGCTCGCTGT
pGMC-63_64_Del_N24aa_Fw:	TAAGAGTGAGCCACCTGCGGCGCATC
pGMC-63_64_Del_N24aa_Rv:	GGTGGCTCACTCTTATCTCATCACCTCAGG
pGMC-63_64_N25aa-C30aa_FW:	ATGCCAGCGGCGACACCTGGGCAGCC

pGMC-63_64_N25aa-C30aa_RV:	TGTCGCCGCTGGCATCGTGGGCGTCG
pGMC-63+64_Del_C28aa_Fw:	CCGGCGACTAACAACTTTATTATACATAGTTGA
pGMC-63+64_Del_C28aa_Rv:	GTTGTTAGTCGCCGGCGGCGCGGGCA
pGMC-63-64*T5A-Fw:	AGAGTGAAGCACAAGGCCGACATTGACGAGTG
pGMC-63-64*T5A-Rv:	CACTCGTCAATGTCGGCCTTGTGCTTCACTCT
infu_AWB99_RS04535_Fw	CTCGGCAAGGAGGTCCTGATGGCGGGGCAACGCAAC
linear GMC RS04530_Rv	CAGGACCTCCTTGCCGAGCGGAG
**Toxin and antitoxin cloning in pLAM**	
pLAM-Rv2663_Fw:	GACATATGGAGGTGAGGGCTAGC (NdeI)
pLAM-Rv2663_Rv:	GAGAATTCTCACCTCAGGTAGTC (EcoRI)
pLAM-Rv2664_Fw:	GACATATGAAGCACAAGACCGAC (NdeI)
pLAM-Rv2664_Rv:	GAGAATTCTTAGGGGCTCGCTGT (EcoRI)
pLAM12-Rv2664-infu-Fw:	AGAAAGGGAGTCCACATATGAAGCACAAGAC
pLAM12-Rv2664-infu-RV:	ATAAGCTTCGAATTCTTAGGGGCTCGCTGT
pLAM12-infu-Fw:	ATGTGGACTCCCTTTCTCTTATC
pLAM12-infu-Rv:	GAATTCGAAGCTTATCGATG
pLAM12-Rv2664-delN2-24:	AGGGAGTCCACATATGAGCCACCTGCGGCGCATC
pLAM12-Rv2664-delC57-84:	ATAAGCTTCGAATTCTTAGTCGCCGGCGGCGCGGGCA
Rv2664_D6A_Fw:	TGAAGCACAAGACCGCCATTGACGAGTGGCTC
Rv2664_D6A_Rv:	GAGCCACTCGTCAATGGCGGTCTTGTGCTTCA
Rv2664_D8A_Fw:	ACAAGACCGACATTGCCGAGTGGCTCGACAC
Rv2664_D8A_Rv:	GTGTCGAGCCACTCGGCAATGTCGGTCTTGT
Rv2664_W10A_Fw:	ACCGACATTGACGAGGCGCTCGACACGATCGAG
Rv2664_W10A_Rv:	CTCGATCGTGTCGAGCGCCTCGTCAATGTCGGT
Rv2664_D12A_Fw:	TTGACGAGTGGCTCGCCACGATCGAGCCCAAC
Rv2664_D12A_Rv:	GTTGGGCTCGATCGTGGCGAGCCACTCGTCAA
Rv2664_H22A_Fw:	AACCCGGCCGACGCCGCCGATGCCAGCCACCT
Rv2664_H22A_Rv:	AGGTGGCTGGCATCGGCGGCGTCGGCCGGGTT
Rv2664_R29A_Fw:	TGCCAGCCACCTGCGGGCCATCATCGCCGCGA
Rv2664_R29A_Rv:	TCGCGGCGATGATGGCCCGCAGGTGGCTGGCA
Rv2664_E35A_Fw:	TCATCGCCGCGAAAGCAGCGGTCCAAACAG
Rv2664_E35A_Rv:	CTGTTTGGACCGCTGCTTTCGCGGCGATGA
Rv2664_E43A_Fw:	CAAACAGCCGAATCTGCGTTGCGGGCCGCAGT
Rv2664_E43A_Rv:	ACTGCGGCCCGCAACGCAGATTCGGCTGTTTG
Rv2664_V63A_Fw:	TGGGCAGCCATCGGCGCCGCCCTCGGCATCAC
Rv2664_V63A_Rv:	GTGATGCCGAGGGCGGCGCCGATGGCTGCCCA
Rv2664_R69A_Fw:	TGGGCAGCCATCGGCGCCGCCCTCGGCATCAC
Rv2664_R69A_Rv:	GTGATGCCGAGGGCGGCGCCGATGGCTGCCCA
Rv2664_D6A/D8A_Fw:	ACAAGACCGCCATTGCCGAGTGGCTCGACAC

Rv2664_D6A/D8A_Rv:	GTGTCGAGCCACTCGGCAATGGCGGTCTTGT
Rv2664_D6A/D8A/D12A_Fw:	CATTGCCGAGTGGCTCGCCACGATCGAGCCCAA
Rv2664_D6A/D8A/D12A_Rv:	TTGGGCTCGATCGTGGCGAGCCACTCGGCAATG
pLAM- RS04530_Fw	GACATATGCAGCTGAACAGGCCGTTC (NdeI)
pLAM- RS04530_Rv	GAGAATTCTCAGGACCTCCTTGCCGAG (EcoRI)
pLAM- RS04535_Fw	GACATATGGCGGGGCAACGCAACTGTG (NdeI)
pLAM- RS04535_Rv	GAGAATTCCTACCCCCGAGCGGCCACTG (EcoRI)
**Toxin cloning in pMPMK6**	
Rv2663-pMPMK6-ligation_Fw:	GAGAATTCCATATGGAGGTGAGGGCTAGCGC (EcoRI)
Rv2663-pMPMK6-ligation_Rv:	GAAAGCTTGGATCCTCACCTCAGGTAGTCGTAGA (HindIII)
**Antitoxin cloning in p29SEN**	
Rv2664-p29SEN-ligation_Fw:	GAGAATTCCATATGAAGCACAAGACCGACAT (EcoRI)
Rv2664-p29SEN-ligation_Rv:	GAAAGCTTGGATCCTTAGGGGCTCGCTGTGCTGT (HindIII)
**Toxin cloning in pET vector**	
pET20b-Rv2663_Liga_Fw:	GACATATGGAGGTGAGGGCTAGCGCCCGCAA (NdeI)
pET20b-Rv2663_Liga_Rv:	GACTCGAGCCTCAGGTAGTCGTAGAACTT (XhoI)
15b- RelI NdeI For	TTCATATGAAGCACAAGACCGACATTG (NdeI)
15b- RelI del 2-24 For	TTCATATGAGCCACCTGCGGCGCATC (NdeI)
15b- RelI BamHI Rv	TTGGATCCTTAGGGGCTCGCTGTGCTG (BanHI)
**PURE T7 DNA template synthesizing**	
PURE cspA_Fw:	GCGAATTAATACGACTCACTATAGGGCTTAAGTATAAGGAGGAAAAAATATGCCACAGGGAACTGTGAAG
PURE cspA_Rv:	AAACCCCTCCGTTTAGAGAGGGGTTATGCTAGTCAGAGGGAGCGGACTCCGGTGGCCTG
PURE gfp_Fw:	GCGAATTAATACGACTCACTATAGGGCTTAAGTATAAGGAGGAAAAAATATGGAGTAAAGGAGAAGAACTTTTCACTG
PURE gfp_Rv:	AAACCCCTCCGTTTAGAGAGGGGTTATGCTAGTTATTTGTAGAGCTCATCCATGCCATGTG
**Extension primers**	
cspA extension primer-1:	TCTCCGTGTAGTGGACAAATACATCCGCGGAAC
cspA extension primer-2:	AGCGAGCGGACTCCGGTGGCCT
gfp extension primer-1:	AGGGTAAGTTTTCCGTATGTTGCATCACCTTCAC
gfp extension primer-2:	TCTGCTAGTTGAACGGATCCATCTTCAATGTTGTG
**Northern Blot probes**	
tRNA probes: (Cai *et al*, 2020)	
*M.smeg*_Arg1_tRNA_probe:	TGCGCCCGAAGGGATTCGAA
*M.smeg*_Cys1_tRNA_probe:	TCGAGCGAGTGACGGGACTC
*M.smeg*_Met2_tRNA_probe:	TAGCGGTGGGGGGGCTCGAT
*M.smeg*_Thr1_tRNA_probe:	AGCCGCCTGGGGGAATCGAA

*M.smeg*_Sec_tRNA_probe:	TGGCGGAGGCGGACGGGAAT
cspA_mRNA_3'end_probe:	AGCGAGCGGACTCCGGTGGCCT
gfp_mRNA _3'end_probe:	ATTTGTAGAGCTCATCCATGCCA
*M.smeg*_5SrRNA_3'end_probe:	AGTATCATCGGCGCTGGCAG
*M.smeg*_16SrRNA_3'end_probe:	AGAAAGGAGGTGATCCAGCC
*M.smeg*_16SrRNA_Middle_probe:	CTGTTCGCTCCCCACGCTTT
*M.smeg*_23SrRNA_3'end_probe:	GTAAGTTTTCGGCCGGTTAG
**RNase H digestion and 3'OH sequence library**	
16S_RNaseH_ probe_1:	ACGUAUUCACCGC<u>AGCGTT</u>GCUGAUCUGCGAUUAC
3P_V4 primer:	GTATCTNNNNNNNNNNNNNNNNTGAGCCTCGGTTGGTGCCG
D6A:	CTCTTTCCCTACACGACGCTCTTCCGATCTNTACACGGCACCAACCGAGG
D6B:	CTCTTTCCCTACACGACGCTCTTCCGATCTNGTATCGGCACCAACCGAGG
D6C:	CTCTTTCCCTACACGACGCTCTTCCGATCTNCGTCCGGCACCAACCGAGG
D6D:	CTCTTTCCCTACACGACGCTCTTCCGATCTNAAGTCGGCACCAACCGAGG
D6E:	CTCTTTCCCTACACGACGCTCTTCCGATCTNACACCGGCACCAACCGAGG
D6F:	CTCTTTCCCTACACGACGCTCTTCCGATCTNGGTACGGCACCAACCGAGG
D6H:	CTCTTTCCCTACACGACGCTCTTCCGATCTNTCGGCGGCACCAACCGAGG
D6I:	CTCTTTCCCTACACGACGCTCTTCCGATCTNCAAGCGGCACCAACCGAGG
D6J:	CTCTTTCCCTACACGACGCTCTTCCGATCTNTTGACGGCACCAACCGAGG
D6K:	CTCTTTCCCTACACGACGCTCTTCCGATCTNGCTGCGGCACCAACCGAGG
D6L:	CTCTTTCCCTACACGACGCTCTTCCGATCTNCCGACGGCACCAACCGAGG
D6M:	CTCTTTCCCTACACGACGCTCTTCCGATCTNCTCGCGGCACCAACCGAGG
*M.smeg*_16SrRNA_RNAseq_3:	CTGGAGTTCAGACGTGTGCTCTTCCGATCT TGAATACGTTCCCGGGCCTT
A-PE-PCR10:	AATGATACGGCGACCACCGAGATCTACACTCTTTCCCTACACGACG
B_i7RPI1_CGTGAT:	CAAGCAGAAGACGGCATACGAGATCGTGATGTGACTGGAGTTCAGACGTGTGC
B_i7RPI2_ACATCG:	CAAGCAGAAGACGGCATACGAGATACATCGGTGACTGGAGTTCAGACGTGTGC
B_i7RPI3_GCCTAA:	CAAGCAGAAGACGGCATACGAGATGCCTAAGTGACTGGAGTTCAGACGTGTGC

Supplementary Table S2: List of plasmids used in this work

Name			References
Empty vectors			
pJV53			(1)
pGMC			(2)
pLAM12			(1)
pMPMK6			(3)
p29SEN			(4)
pET20b			Novagen
pET15b			Novagen
pTRB550			(5)
pTA100			(6)
**Toxins/Antitoxins wild-type and mutants**			
**Name**	**Specificity**	**Backbone**	**References**
pGMC-RelS	*M. tuberculosis* Rv2663	pGMC	This study
pGMC-RelI	*M. tuberculosis* Rv2664	pGMC	This study
pGMC-AWB99_RS04530	*M. confluentis* AWB99_RS04530	pGMC	This study
pGMC-AWB99_RS04535	*M. confluentis* AWB99_RS04535	pGMC	This study
pGMC-Mycch_5153	*M. chubuense* Mycch_5153	pGMC	This study
pGMC-Mycch_5154	*M. chubuense* Mycch_5154	pGMC	This study
pGMC-BST38_RS17710	*M. parafortuitum* BST38_RS17710	pGMC	This study
pGMC-BST38_RS29380	*M. parafortuitum* BST38_RS29380	pGMC	This study
pGMC-BLW81_RS13130	*M. rutilum* BLW81_RS13130	pGMC	This study
pGMC-BLW81_RS13135	*M. rutilum* BLW81_RS13135	pGMC	This study
pGMC-AFA91_01820	*M. goodii* AFA91_01820	pGMC	This study
pGMC- AFA91_01825	*M. goodii* AFA91_01825	pGMC	This study
pGMC-Mycch_5672	*M. chubuense* Mycch_5672	pGMC	This study
pGMC-Mycch_5673	*M. chubuense* Mycch_5673	pGMC	This study
pGMC-AWB99_RS04530-RS04535	M. confluentis AWB99_RS04530-RS04535	pGMC	This study
pGMC-WSD-RelS	*M. tuberculosis* Rv2663	pGMC	This study
pGMC-WSD-RelS*R4A	*M. tuberculosis* Rv2663 R6A	pGMC	This study
pGMC-WSD-RelS*S6A	*M. tuberculosis* Rv2663 S6A	pGMC	This study
pGMC-WSD-RelS*K9A	*M. tuberculosis* Rv2663 K9A	pGMC	This study
pGMC-WSD-RelS*H10A	*M. tuberculosis* Rv2663 H10A	pGMC	This study
pGMC-WSD-RelS*L25A	*M. tuberculosis* Rv2663 L25A	pGMC	This study
pGMC-WSD-RelS*Y27A	*M. tuberculosis* Rv2663 Y27A	pGMC	This study
pGMC-WSD-RelS*E31A	*M. tuberculosis* Rv2663 E31A	pGMC	This study
pGMC-WSD-RelS*Y32A	*M. tuberculosis* Rv2663 Y32A	pGMC	This study
pGMC-WSD-RelS*H33A	*M. tuberculosis* Rv2663 H33A	pGMC	This study
pGMC-WSD-RelS*T46A	*M. tuberculosis* Rv2663 T46A	pGMC	This study
pGMC-WSD-RelS*R48A	*M. tuberculosis* Rv2663 R48A	pGMC	This study
pGMC-WSD-RelS*R61A	*M. tuberculosis* Rv2663 R61A	pGMC	This study
pGMC-WSD-RelS*H64A	*M. tuberculosis* Rv2663 H64A	pGMC	This study
pGMC-WSD-RelS*R69A	*M. tuberculosis* Rv2663 R69A	pGMC	This study
pGMC-WSD-RelS*K71A	*M. tuberculosis* Rv2663 K71A	pGMC	This study
pGMC-WSD-RelS $^{\text{His tag}}$	*M. tuberculosis* Rv2663-His Tag	pGMC	This study
pGMC-WSD-RelS*H10A $^{\text{His tag}}$	*M. tuberculosis* Rv2663 H10A-His Tag	pGMC	This study
pGMC-RelS-RelI	*M. tuberculosis* Rv2663-Rv2664	pGMC	This study
pGMC-RelS-RelI *T5A	*M. tuberculosis* Rv2663-Rv2664 T5A	pGMC	This study

pGMC-RelS-RelI *D6A	*M. tuberculosis* Rv2663-Rv2664 D6A	pGMC	This study
pGMC-RelS-RelI *D8A	*M. tuberculosis* Rv2663-Rv2664 D8A	pGMC	This study
pGMC-RelS-RelI *W10A	*M. tuberculosis* Rv2663-Rv2664 W10A	pGMC	This study
pGMC-RelS-RelI *D12A	*M. tuberculosis* Rv2663-Rv2664 D12A	pGMC	This study
pGMC-RelS-RelI *H22A	*M. tuberculosis* Rv2663-Rv2664 H22A	pGMC	This study
pGMC-RelS-RelI *R29A	*M. tuberculosis* Rv2663-Rv2664 R29A	pGMC	This study
pGMC-RelS-RelI *E35A	*M. tuberculosis* Rv2663-Rv2664 E35A	pGMC	This study
pGMC-RelS-RelI *E43A	*M. tuberculosis* Rv2663-Rv2664 E43A	pGMC	This study
pGMC-RelS-RelI *R69A	*M. tuberculosis* Rv2663-Rv2664 R69A	pGMC	This study
pGMC-RelS-RelI *D6A/D8A	*M. tuberculosis* Rv2663-Rv2664 D6A/D8A	pGMC	This study
pGMC-RelS-RelI *D6A/D12A	*M. tuberculosis* Rv2663-Rv2664 D6A/D12A	pGMC	This study
pGMC-RelS-RelI *D8A/D12A	*M. tuberculosis* Rv2663-Rv2664 D8A/D12A	pGMC	This study
pGMC-RelS-RelI *D8A/E35A	*M. tuberculosis* Rv2663-Rv2664 D8A/E35A	pGMC	This study
pGMC-RelS-RelI *D8A/E43A	*M. tuberculosis* Rv2663-Rv2664 D8A/E43A	pGMC	This study
pGMC-RelS-RelI *D8A/R69A	*M. tuberculosis* Rv2663-Rv2664 D8A/R69A	pGMC	This study
pGMC-RelS-RelI *D6A/D8A/D12A	*M. tuberculosis* Rv2663-Rv2664 D6A/D8A/D12A	pGMC	This study
pGMC-RelS-Δ(2-24)RelI	*M. tuberculosis* Rv2663-Rv2664 Δ2-24	pGMC	This study
pGMC-RelS-Δ(26-54)RelI	*M. tuberculosis* Rv2663-Rv2664 Δ25-54	pGMC	This study
pGMC-RelS-Δ(57-84)RelI	*M. tuberculosis* Rv2663-Rv2664 Δ57-84	pGMC	This study
pLAM-RelS	*M. tuberculosis* Rv2663	pLAM12	This study
pLAM-RelI	*M. tuberculosis* Rv2664	pLAM12	This study
pLAM-AWB99_RS04530	*M. confluentis* AWB99_RS04530	pLAM12	This study
pLAM-AWB99_RS04535	*M. confluentis* AWB99_RS04535	pLAM12	This study
pLAM-RelI*D6A	*M. tuberculosis* Rv2664 D6A	pLAM12	This study
pLAM-RelI*D8A	*M. tuberculosis* Rv2664 D8A	pLAM12	This study
pLAM-RelI*W10A	*M. tuberculosis* Rv2664 W10A	pLAM12	This study
pLAM-RelI*D12A	*M. tuberculosis* Rv2664 D12A	pLAM12	This study
pLAM-RelI*H22A	*M. tuberculosis* Rv2664 H22A	pLAM12	This study
pLAM-RelI*R29A	*M. tuberculosis* Rv2664 R29A	pLAM12	This study
pLAM-RelI*E35A	*M. tuberculosis* Rv2664 E35A	pLAM12	This study
pLAM-RelI*E43A	*M. tuberculosis* Rv2664 E43A	pLAM12	This study
pLAM-RelI*R69A	*M. tuberculosis* Rv2664 R69A	pLAM12	This study
pLAM-RelI*D6A/D8A	*M. tuberculosis* Rv2664 D6A/D8A	pLAM12	This study
pLAM-RelI*D6A/D12A	*M. tuberculosis* Rv2664 D6A/D12A	pLAM12	This study
pLAM-RelI*D8A/E29A	*M. tuberculosis* Rv2664 D8A/E29A	pLAM12	This study
pLAM-RelI*D8A/E35A	*M. tuberculosis* Rv2664 D8A/E35A	pLAM12	This study
pLAM-RelI*D8A/E43A	*M. tuberculosis* Rv2664 D8A/E43A	pLAM12	This study
pLAM-RelI*D8A/V63A	*M. tuberculosis* Rv2664 D8A/V63A	pLAM12	This study
pLAM-RelI*D8A/R69A	*M. tuberculosis* Rv2664 D8A/R69A	pLAM12	This study
pLAM-RelI*R29A/E43A	*M. tuberculosis* Rv2664 R29A/E43A	pLAM12	This study
pLAM-RelI*E35A/V63A	*M. tuberculosis* Rv2664 E35A/V63A	pLAM12	This study
pLAM-RelI*E35A/R69A	*M. tuberculosis* Rv2664 E35A/R69A	pLAM12	This study
pLAM-RelI*D6A/D8A/D12A	*M. tuberculosis* Rv2664 D6A/D8A/D12A	pLAM12	This study

pLAM-RelI*D6A/D8A/D12A/E35A/V63A/R69A	*M. tuberculosis* Rv2664 D6A/D8A/D12A/E35A/V63A/R69A	pLAM12	This study
pLAM-Δ(2-24)RelI	*M. tuberculosis* Rv2664 Δ2-24	pLAM12	This study
pLAM-Δ(26-54)RelI	*M. tuberculosis* Rv2664 Δ25-54	pLAM12	This study
pLAM-Δ(57-84)RelI	*M. tuberculosis* Rv2664 Δ57-84	pLAM12	This study
pMPMK6-RelS	*M. tuberculosis* Rv2663	pMPMK6	This study
pMPMK6-RelS*H10A	*M. tuberculosis* Rv2663 H10A	pMPMK6	This study
p29SEN-RelI	*M. tuberculosis* Rv2664	p29SEN	This study
pET20b-RelS	*M. tuberculosis* Rv2663-His Tag	pET20b	This study
pET20b-RelS*H10A	*M. tuberculosis* Rv2663 H10A-His Tag	pET20b	This study
pTRB686 (His-SUMO-RelS)	*M. tuberculosis* Rv2663	pTRB550	This study
pTRB715 (His-SUMO-RelS H10A)	*M. tuberculosis* Rv2663 H10A	pTRB550	This study
pTRB695	*M. tuberculosis* Rv2664	pTA100	This study
pTRB743	*M. tuberculosis* Rv2664 Δ2-24	pTA100	This study
pET15b-RelI	*M. tuberculosis* Rv2664	pET15b	This study
pET15b-Δ(2-24)RelI	*M. tuberculosis* Rv2664 Δ2-24	pET15b	This study

References

1. Kessel,J.C. van and Hatfull,G.F. (2007) Recombineering in Mycobacterium tuberculosis. *Nat Methods*, **4**, 147–152.
2. Blumenthal,A., Trujillo,C., Ehrt,S. and Schnappinger,D. (2010) Simultaneous Analysis of Multiple Mycobacterium tuberculosis Knockdown Mutants In Vitro and In Vivo. *PLOS ONE*, **5**, e15667.
3. Mayer,M.P. (1995) A New Set of Useful Cloning and Expression Vectors Derived from Pbluescript. *Gene*, **163**, 41–46.
4. Genevaux,P., Keppel,F., Schwager,F., Langendijk-Genevaux,P.S., Hartl,F.U. and Georgopoulos,C. (2004) In vivo analysis of the overlapping functions of DnaK and trigger factor. *EMBO Rep.*, **5**, 195–200.
5. Cai,Y., Usher,B., Gutierrez,C., Tolcan,A., Mansour,M., Fineran,P.C., Condon,C., Neyrolles,O., Genevaux,P. and Blower,T.R. (2020) A nucleotidyltransferase toxin inhibits growth of Mycobacterium tuberculosis through inactivation of tRNA acceptor stems. *Sci Adv*, **6**, eabb6651.
6. Fineran,P.C., Blower,T.R., Foulds,I.J., Humphreys,D.P., Lilley,K.S. and Salmond,G.P.C. (2009) The phage abortive infection system, ToxIN, functions as a protein-RNA toxin-antitoxin pair. *Proc Natl Acad Sci U S A*, **106**, 894–899.
